# Determinants of SARS-CoV-2 outcomes in patients with cancer vs controls without cancer: a multivariable meta-analysis with genomic imputation

**DOI:** 10.1016/j.eclinm.2025.103194

**Published:** 2025-05-02

**Authors:** Mark T.K. Cheng, James S. Morris, Syed F.H. Shah, Abraham Tolley, José Chen-Xu, Nihal Sogandji, Long H. Fong, Anushka Irodi, Justine T.N. Chan, Kimia Kamelian, Benjamin L. Sievers, Shazia Sarela, Margaret K. Ho, Abigail Burn, Anita Patel, Ghislaine D. Mbolo, Muhammad Hasan, Abdulbasit O. Fehintola, Chan C. Yin, Enti Spata, Ravindra K. Gupta, David M. Favara

**Affiliations:** a University of Cambridge School of Clinical Medicine, Addenbrooke's Hospital NHS Foundation Trust, Hills Road, Cambridge, UK, CB2 0QQ; b Cambridge Institute of Therapeutic Immunology & Infectious Disease (CITIID), Department of Medicine, University of Cambridge, Cambridge, UK; c John Radcliffe Hospital, Oxford University Hospitals NHS Foundation Trust, Oxford, UK; d National School of Public Health, NOVA University of Lisbon, Portugal; e Public Health Unit, Local Health Unit Baixo Mondego, Figueira da Foz, Portugal; f Department of Health Policy, London School of Economics and Political Science, London, WC2A 2AE, UK; g University of Cambridge Department of Medicine, Addenbrooke's Hospital NHS Foundation Trust, Hills Road, Cambridge, UK, CB2 0QQ; h Department of Medicine, University College London (UCL), London, UK; i Department of Medicine, Queen Mary Hospital, Hong Kong; j School of Medicine, University of Liverpool, Liverpool, UK; k Leeds School of Medicine, University of Leeds, Leeds, UK; l College of Medicine, University of Ibadan, Ibadan, Nigeria; m Li Ka Shing Faculty of Medicine, The University of Hong Kong, Hong Kong Special Administrative Region of China; n Biometrics, Respiratory and Immunology, Research and Development, AstraZeneca, Cambridge, UK; o MRC-Laboratory of Molecular Biology, Cambridge, UK; p Department of Oncology, University of Cambridge, Cambridge, UK; q Department of Oncology, Cambridge University Hospitals NHS Foundation Trust, Hills Road, Cambridge, UK, CB2 0QQ

**Keywords:** SARS-CoV-2, COVID-19, Cancer, Meta-analysis, Systematic review, Mortality, Intensive care, Severity, Hospitalisation

## Abstract

**Background:**

SARS-CoV-2 is known to impact patients with cancer adversely. Previous meta-analyses have lacked clarity on the recency of cancer diagnosis, anti-cancer treatment durations, and SARS-CoV-2 specific variants of concern (VOC). This study aimed to compare SARS-CoV-2 multivariable-adjusted clinical outcomes between patients with cancer and those without cancer, identifying key risk factors spanning pre- and post-Omicron periods.

**Methods:**

In this systematic review and meta-analysis, we identified from Medline, Embase, Cochrane Central, and the WHO COVID-19 Research Database prospective and retrospective case–control studies and cohort studies published from 1st January 2019 to 22nd November 2024. We included case–control and cohort studies comparing at least 10 patients with active cancer (diagnosed or treated within three years prior to SARS-CoV-2 infection) to controls without cancer using multivariable analyses. Exclusion criteria included lack of clarity about active/inactive status of cancer, lack of a control group without cancer, lack of multivariate analysis comparing outcomes of interest in patients with active cancer vs patients without cancer, case reports or case series, and SARS-CoV-2 diagnosis not confirmed via laboratory testing. Outcomes measured were SARS-CoV-2 infection severity (WHO ordinal scale) and mortality differences by tumour type, treatment, and VOC (using sequencing data from NCBI Genbank and GISAID). A random-effects meta-analysis model was applied. The systematic review was PRISMA compliant and was registered with PROSPERO, CRD420234454524.

**Findings:**

Of 35,501 studies initially identified, 30 met eligibility criteria and were included in the meta-analysis, comprising 281,270 patients with cancer and 18,876,411 controls. Using the Agency for Healthcare Research and Quality (AHRQ) risk of bias standards, 21 studies were rated good, one study rated was fair, and eight studies were rated poor. We found higher mortality odds ratios (OR) in patients with cancer infected with SARS-CoV-2: 1·40 (95% CI: 1·12–1·73, I^2^ = 98·1%) for solid tumours and 2·10 (95% CI: 1·43–3·07, I^2^ = 97·3%) for haematological malignancies, with the difference in mortality between these groups not reaching statistical significance (Q (1) = 3·32; *p* = 0·0068). Amongst the solid cancers, thoracic and colorectal were linked to increased odds of mortality (ORs: 2·63 [95% CI: 1·65–4·20, I^2^ = 98·7%], and 1·65 [95% CI: 1·26–2·15, I^2^ = 92·7%], respectively). Metastatic cancers (OR: 3·59; 95% CI: 1·07–12·04, I^2^ = 99·5%) were also linked to greater odds of mortality compared to localised cancers (OR: 1·76; 95% CI: 1·32–2·34, I^2^ = 96·6%; *p* = 0·26). No cancer types showed a reduced risk vs controls. Mortality varied significantly among VOCs; Alpha (OR: 4·59; 95% CI: 2·66–7·92, I^2^: N/A) and Omicron (OR: 2·74; 95% CI: 1·84–4·09, I^2^ = 90·2%) were more associated with death than the ancestral Wu-1 (OR: 1·43; 95% CI: 1·14–1·80, I^2^ = 98·2%) and Delta (OR: 1·94; 95% CI: 1·65–2·29, I^2^:N/A) variants (X^2^ (4) = 20·4; *p* = 0·0004).

**Interpretation:**

This comprehensive meta-analysis indicates that patients with active cancer with SARS-CoV-2 have a higher risk of mortality and hospitalisation than those without cancer. The risk of death was comparable between active solid and haematological tumours. SARS-CoV-2 severity and mortality risks were higher with thoracic, colorectal, or any metastatic cancers. Additionally, differences were noted in mortality risks across VOCs, diverging from VOC-associated mortality patterns in the general population. However, the strict three-year cutoff used to define active cancer excludes studies that used broader cancer criteria (i.e., any history of cancer), which may limit generalisability. Further limitations include varied definitions of disease severity, retrospective data collection, incomplete vaccination or lineage data, and significant between-study heterogeneity, potentially influencing these findings.

**Funding:**

10.13039/501100000289Cancer Research UK; 10.13039/100014013UK Research and Innovation.

**Research in context**

**Evidence before this study**

Prior to this study we searched Medline, Embase, Cochrane, and WHO COVID-19 databases from January 2020 to 12 October 2023, identifying three prior meta-analyses (comprising 19, 35, and 57 studies, respectively) comparing SARS-CoV-2 outcomes in patients with cancer compared to patients without cancer. These analyses consistently showed that patients with cancer experienced poorer outcomes than those without cancer. However, they offered limited or no detail on the recency of cancer diagnosis, cancer treatment status, and were conducted mainly before the emergence of SARS-CoV-2 variants of concerns (VOCs).

**Added value of this study**

To our knowledge, this is the largest meta-analysis to date evaluating outcomes in patients diagnosed with or treated for active cancer within three years prior to their index SARS-CoV-2 case, and includes over 280,000 patients with cancer and more than 18 million controls. Our findings indicate higher mortality odds ratios for both solid and haematological malignancies relative to controls, with no statistically significant difference between these two cancer groups, challenging earlier findings that patients with haematological cancers have poorer SARS-CoV-2 outcomes than patients with solid cancers. Additionally, we leveraged the Global Initiative on Sharing All Influenza Data (GISAID) and National Center for Biotechnology Information (NCBI) Genbank databases for SARS-CoV-2 sequencing data and WHO reported weekly cases and deaths to perform genomic imputation, which was validated using studies which reported dominant VOCs. We found that the odds of mortality between cancer and non-cancer patients of VOCs differed, with Alpha and Omicron having the greatest association with death in patients with active cancer compared to ancestral and Delta variant.

**Implications of all the available evidence**

By clearly defining active cancer status, this study refines risk stratification for patients most vulnerable to severe SARS-CoV-2 disease, though the three-year cutoff for active cancer may be overly restrictive. Nonetheless, our findings to the evidence base that patients with cancer are at a greater odd of SARS-COV-2 related morbidity and mortality than patients without cancer, warranting continuous protective measures. The availability of genomic and epidemiological data is useful for monitoring outcomes in the general population and specific patient groups for SARS-CoV-2, influenza, and future emerging pandemics.

## Introduction

Patients with cancer are known to be at increased risk of mortality from infections like SARS-CoV-2 (COVID-19).^1^, ^2^, ^3^ Previous meta-analyses indicate that patients with cancer face higher risks of mortality with severe SARS-CoV-2 infection,^4^, ^5^, ^6^, ^7^ though responses vary by cancer type and treatment. Our previous systematic review of 110 studies showed a five-fold mortality risk increase in patients with cancer compared to patients without cancer.^6^ The ISARIC WHO CCP UK group found cancer to be a risk factor for mortality, especially in younger patients.^8^^,^^9^

COVID-19 was defined by unprecedented viral evolution, with regular emergence of antigenically distinct immune escape variants,^10^^,^^11^ distinct waves of variant of concerns (VOCs) were able to spread across the globe rapidly and assert global dominance. These variants most likely emerged in immune compromised individuals,^12^ and were characterised by increasing fitness,^13^ immune escape from neutralising antibodies, and indeed change in tissue tropism with the arrival of Omicron.^14^

The epidemiological pattern of SARS-CoV-2 VOCs is similar to the cyclical nature of yearly influenza seasons, where waves of variants and strains are antigenically distinct, transmit at varying rates, and have different severity profiles.^15^ While common for the influenza virus to be subtyped via PCR as part of routine clinical practice,^16^ SARS-CoV-2 variants were only distinguished by S-gene target failure (SGTF) or sequencing.^17^ Thus, SARS-CoV-2 clinical severity studies often do not report a dominant variant, or define the dominant variant by the study period.^8^^,^^18^ The maturation of pathogen sequence databases such as NCBI Genbank^19^^,^^20^ and GISAID,^21^^,^^22^ has enabled improved epidemiological studies–variant surveillance can happen at real time, and imputation of transmission events^23^ and chronic infection^24^ becomes possible. There is a need to better understand mortality changes for VOCs, and accurate lineage assignment of historical genomic sequences can be leveraged to genomically impute the predominant variant in studies. Recent studies suggest that despite vaccination and evolving SARS-CoV-2 variants and immunity changes, mortality reductions are not uniform, particularly for those with haematological malignancies.^25^, ^26^, ^27^, ^28^

Previous studies may overstate the impact of cancer on COVID-19 outcomes by including patients in remission or not currently on immunocompromising treatment.^4^, ^5^, ^6^, ^7^^,^^29^ Our review aims to discern outcomes for patients with active cancer vs those without cancer and identify high-risk subgroups. We also explore how relative risks have shifted throughout the pandemic, which could inform future research and pandemic responses. To the best of our knowledge, this meta-analysis is the first to focus on multivariate outcome analysis in patients with a cancer diagnosis or treatment history within three years of their SARS-CoV-2 index case.

## Methods

### Search strategy and selection criteria

This systematic review was conducted according to the Preferred Reporting Items for Systematic Review and Meta-Analyses (PRISMA) statement,^30^ and its protocol was registered prospectively under PROSPERO (CRD42023354524). Medline (Ovid), Embase (Ovid), Cochrane Library, and the World Health Organisation (WHO) COVID-19 Research Database were repeatedly searched for peer-reviewed or grey literature from 1st January 2019 to 22nd November 2024. Duplicate studies were identified and removed. Detailed search strategies are shown in Document S1.

The PECOS framework was used to define inclusion criteria. Population: patients with reverse transcription polymerase chain reaction (RT-PCR) or rapid antigen test confirmed SARS-CoV-2 infection; Exposure: patients with active cancer (defined as cancer diagnosis or cancer treatment within three years of index SARS-CoV-2 infection); Control: patients without active cancer or a history of cancer; Outcomes: comparing one or more of all-cause/COVID-19 specific mortality, ICU admission/intubation incidence, SARS-CoV-2 infection severity, and hospital admission relative to all SARS-CoV-2 infection cases using multivariable statistics; Study characteristics: peer-reviewed cohort studies.

A three-year cutoff was decided by reviewing previous literature (Khoury, Felice, Han).^4^^,^^5^^,^^7^ We excluded studies with fewer than ten patients with cancer, case–control, case-series, conference abstracts, correspondences, perspectives, and opinion articles. We also excluded studies that confirmed SARS-CoV-2 infection based solely on radiological findings due to the low specificity of radiological investigations compared to PCR testing, with specificity ranging from 11·1–88·9% for chest X-rays,^31^ and 25–80% for chest CTs,^32^ compared to 97% for PCR.^33^^,^^34^ Detailed inclusion and exclusion criteria are listed in Document S2. Studies were independently screened by two reviewers for inclusion by title/abstract and subsequently by full-text. Conflicts were resolved through discussion with a third, more experienced reviewer.

### Data analysis

Two authors independently extracted key trial characteristics and main association measures, with extracted information entered into a customised database designed using Covidence.^35^ SARS-CoV-2 infection outcomes were extracted and categorised according to the 10-point WHO clinical progression scale^36^: ‘Hospitalisation’: hospitalised, no oxygen therapy (score 4); ‘Severe’: hospitalised, oxygen by mask or nasal prongs (score 5); or hospitalised, oxygen by non-invasive ventilation or high flow (score 6); ‘ICU admission’: intubation and mechanical ventilation, pO2/FiO2 ≥ 150 or SpO 2/FiO2 ≥ 200 (score 7); or intubation and mechanical ventilation pO2/FiO2 < 150 (SpO2/FiO2 < 200) or vasopressors (score 8); or intubation and mechanical ventilation pO2/FiO2 < 150 and vasopressors, dialysis, or ECMO (score 9); ‘Mortality’: dead (score 10).

Where studies reported composite rather than individual clinical outcomes, these were included in the meta-analysis under the applicable category of least severity (e.g. composite outcome of ‘ICU admission and/or death’ would be included in the meta-analysis for ‘ICU admission’ only).

Quality assessment was performed using the Newcastle–Ottawa Quality Assessment scale for cohort and case–control studies and subsequent classification according to Agency for Healthcare Research and Quality (AHRQ) standards (Table S1).^37^^,^^38^ Thresholds for converting Newcastle–Ottawa Assessment scales to AHRQ standards (good, fair, poor) are summarised as following: ‘Good’: 3 or 4 points in selection domain AND 1 or 2 points in comparability domain AND 2 or 3 points in outcome/exposure domain; ‘Fair’: 2 points in selection domain AND 1 or 2 points in comparability domain AND 2 or 3 points in outcome/exposure domain; ‘Poor’: 0 or 1 points in selection domain OR 0 points in comparability domain OR 0 or 1 point in outcome/exposure domain.

The National Center for Biotechnology Information (NCBI) GenBank,^19^ and Global Initiative on Sharing All Influenza Data (GISAID),^21^ databases were queried for all SARS-CoV-2 sequences collected from countries associated with a clinical study included in our meta-analysis. The dates searched corresponded to the specific time period of each study per involved country. NCBI GenBank was queried using the LAPIS API,^20^ and GISAID was queried using the Outbreak.info R API.^22^^,^^39^ Subclades of WHO-assigned Variants of Concern (VOCs) were assigned their WHO name and Nextstrain-assigned clade (Table S2).^40^ To account for the changing number of SARS-CoV-2 cases over the study period, we weighed the number of sequences attributed to each VOC by weekly new cases reported to the WHO (https://data.who.int/dashboards/covid19/data):

$${Prevelance}_{\left(variant,study\right)}=\frac{{New\;Cases}_{\left(variant,study\right)}}{{New\;Cases}_{\left(total,study\right)}}$$

Where the weighted new cases attributed to a variant is derived by the weekly summation:

$${New\;Cases}_{\left(variant,study\right)}=\sum_{cw}\left(\frac{{Sequences}_{\left(variant,cw\right)}}{{Sequences}_{\left(total,cw\right)}}\times {New\;cases}_{\left(cw\right)}\right)$$

where cw is the country-week within the defined geographic and time period of each study. If imputed cases of a particular VOC exceeded 50% of total cases within a country during the same timeframe, the study was categorized under the epidemic phase of that VOC.

For each included study, we recorded the odds ratios (ORs) and hazard ratios (HRs) of mortality, ICU admission, severe SARS-CoV-2 infection, and hospitalisation between the patients with cancer cohort and controls without cancer. All statistical tests and *p* values were two-sided and are presented without adjustment for multiple testing. The main analyses employed a random-effects meta-analysis model,^41^^,^^42^ using restricted maximum-likelihood estimates of between-study heterogeneity,^43^ to estimate the overall mean effect size and 95% confidence interval for each reported statistic (OR or HR). A sensitivity analysis of the effect estimates was conducted using a fixed-effect meta-analysis model.^41^ Where data availability allowed, exploratory analyses were conducted to analyse effect estimates by cancer subtype, stage, vaccination status and treatment modality, as well as by predominant SARS-CoV-2 variant. The between-study heterogeneity was assessed using the *p* value of the Cochran's Q-statistic.^44^ Funnel plots were visually inspected to assess how the effect sizes reported by larger studies (at the apex of the funnel) compared with those reported by smaller studies (at its base). In addition, the degree of asymmetry in these funnel plots was evaluated through interpretation of Egger's statistic. All statistical analyses were conducted in Stata version 18·0 Standard Edition (StataCorp LLC, Texas).

### Role of the funding source

The funders had no role in study design, data collection, data analysis, data interpretation, writing of the report, or the decision to submit the manuscript for publication. All authors had full access to the data in the study. All authors had final responsibility for the decision to submit for publication.

## Results

**Fig. 1:**
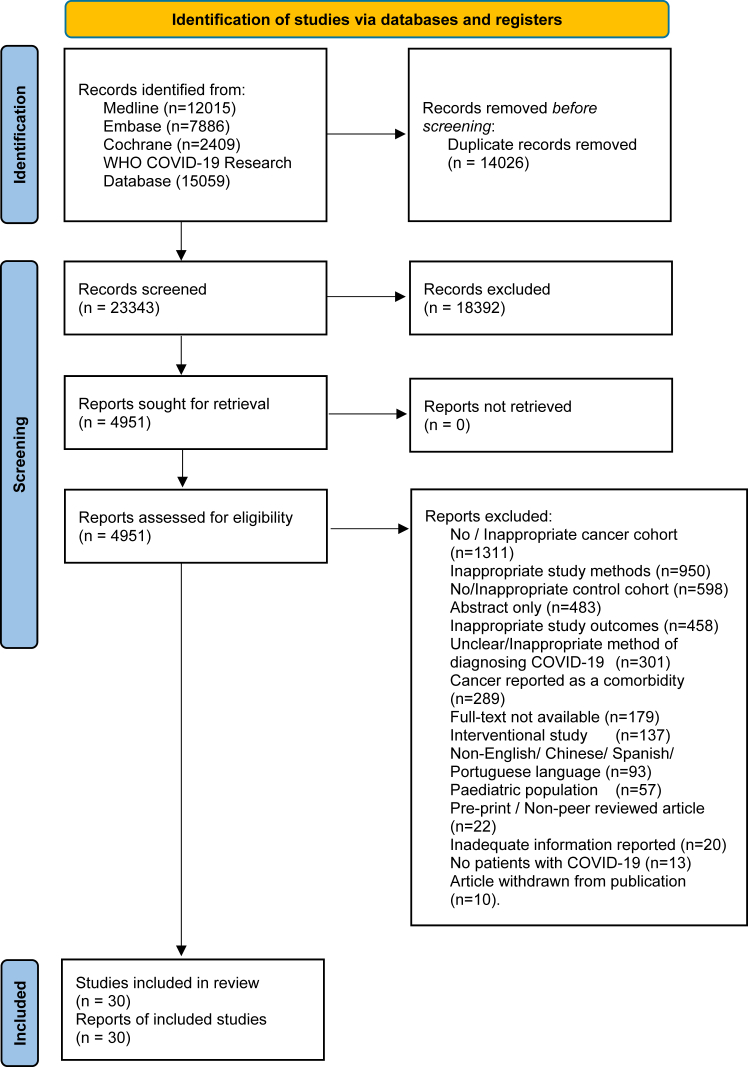
**PRISMA Flow diagram for study selection**. Flow diagram of the systematic review study selection process reported as per PRISMA 2020, mapping out number of records identified, included, excluded, and reasons of exclusions.

**Fig. 2:**
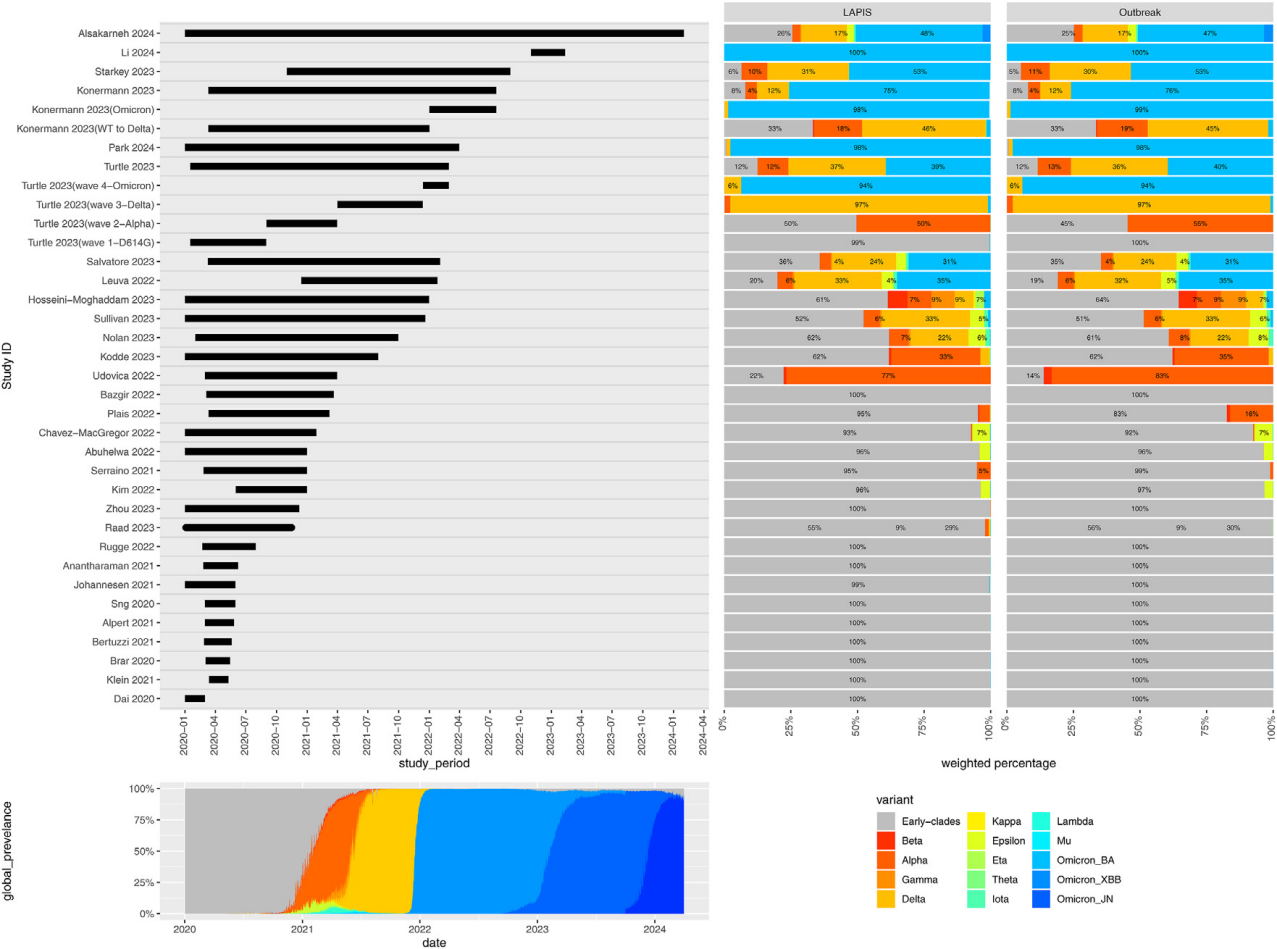
**Dominant variant from epidemiological data**. Temporal distribution (top left) of included studies (*n* = 30), incidence-weighted lineage distribution in the respective study area and time period (top right), and global prevalence dynamics (bottom left) of major variants. A majority of included studies were imputed as pre-Alpha Early-clades due to their early study period.

The PRISMA flowchart detailing our study selection process is provided in Fig. 1.^30^ Our initial search retrieved 37,369 articles for review (Document S1), of which 14,026 were duplicates. 23,343 records were screened and 4951 articles were obtained for full-text eligibility assessment. A total of 30 studies comprising 281,270 patients with cancer and 18,876,411 patients without cancer were included in this systematic review, the individual characteristics for which are presented in Table 1. All 30 included studies were cohort studies,^8^^,^^18^^,^^45^, ^46^, ^47^, ^48^, ^49^, ^50^, ^51^, ^52^, ^53^, ^54^, ^55^, ^56^, ^57^, ^58^, ^59^, ^60^, ^61^, ^62^, ^63^, ^64^, ^65^, ^66^, ^67^, ^68^, ^69^, ^70^, ^71^, ^72^ of which 25 (83·3%) were retrospective in design.^45^^,^^46^^,^^48^, ^49^, ^50^, ^51^, ^52^, ^53^, ^54^, ^55^, ^56^, ^57^, ^58^^,^^60^, ^61^, ^62^, ^63^, ^64^, ^65^, ^66^, ^67^^,^^69^, ^70^, ^71^, ^72^ Patient recruitment spanned the period from January 2020 to February 2024 and is presented for each included study in Fig. 2a. Patients were recruited from 17 different countries with a bias towards the Western Hemisphere (Table S3; Figures S1 and S2). 28 studies (93·3%) included patients who received a cancer diagnosis or cancer treatment within one year of the index SARS-CoV-2 infection, while Leuva et al., 2022 (10,355 patients with cancer)^68^ and Salvatore et al., 2023 (6143 patients with cancer)^67^ instead employed cut-offs of two years and three years, respectively. This contrasts with previous meta-analyses (Tables S4 and S6), in which the majority of included studies comprised patients with cancer without any consideration for the recency of their diagnosis at the time of their index SARS-CoV-2 case.^8^^,^^18^^,^^45^, ^46^, ^47^, ^48^, ^49^, ^50^, ^51^, ^52^, ^53^, ^54^, ^55^, ^56^, ^57^, ^58^, ^59^, ^60^, ^61^, ^62^, ^63^, ^64^, ^65^, ^66^, ^67^, ^68^

**Table 1:** Summary characteristics.

Reference	Study Design	Retrospective/Prospective	Setting	Number of patients			Cancer patients characteristics			Control patients characteristics		AHRQ quality assessment score
				Patients with SARS-CoV-2 and cancer	Patients with SARS-CoV-2 without cancer	Total	Cancer types	Definition of active cancer	Active treatment status	Source	Matching strategy (ratio; variables matched for)	
Alsakarneh 2024	Cohort	Retrospective	NR	15,416	15,416	30,832	Colorectal cancer	ICD-10 clinical modification codes of colorectal cancer (C18–C20).	Included patients on active treatment.	Same national dataset.	1:1; Age, sex, race, ethnicity, comorbidities related to SARS-CoV-2 morbidity and mortality, social determinants of adverse health outcomes, behavioural factors (tobacco smoking, alcohol abuse), COVID vaccine type, cancer treatment types.	Good
Li 2024	Cohort	Retrospective	Inpatient	77	1788	1865	All Types excluding localized skin cancers and early-stage cancer.	Active cancer or those diagnosed with cancer within 1 year of developing community-acquired COVID-19 pneumonia.	Included patients on active treatment.	Same multi-province hospital cluster.	1:3; Age, Acute Physiology and Chronic Health Evaluation (APACHE) II score.	Good
Starkey 2023	Cohort	Retrospective	Inpatient and outpatient	127,322	15,801,004	15,928,326	All Types	Patients in a national cancer registry.	Included patients on active treatment.	Same national dataset.	No matching.	Good
Konermann 2023	Cohort	Prospective	Inpatient	1513	24,912	26,425	All Types	Active cancer based on relavant ICD codes.	NR.	Same national health data repository.	No matching.	Good
Park 2024	Cohort	Retrospective	Inpatient and outpatient	40,334	397,050	437,384	All Types	First ICD-10 cancer diagnosis code (C00–99) <1 year before SARS-CoV-2 diagnosis.	NR.	Same national dataset.	1:1; Age, sex, vaccination against SARS-CoV-2, household income.	Good
Turtle 2023	Cohort	Prospective	Inpatient	5116	134,598	139,714	All Types	Active cancer diagnosis and treatment.	All patients on active treatment.	Same hospital cluster.	No matching.	Good
Salvatore 2023	Cohort	Retrospective	Inpatient and outpatient	6143	38,267	44,410	Bladder, breast, colorectal, kidney, lung, melanoma, prostate, haematological.	Most recent cancer diagnosis within the last 3 years.	Included patients on active treatment.	Same hospital cluster.	No matching.	Poor
Leuva 2022	Cohort	Prospective	Inpatient	10,355	10,696	21,051	All Types	Active diagnosis of new or previously established cancer in the last 2 years.	NR	Same hospital cluster.	1:1; Age, gender, race, body mass index, Elixhauser comorbidity index, vaccination status.	Good
Hosseini-Moghaddam 2023	Cohort	Retrospective	Outpatient	8378	456,196	464,574	All Types	Cancer treatment in the last 6 months or diagnosis within the last 1-year.	Included patients on active treatment.	Same regional health database.	No matching.	Good
Sullivan 2023	Cohort	Retrospective	Inpatient and outpatient	2200	22,000	24,200	Breast cancer	At least two ICD codes for cancer within 1 year before, and anticancer treatment within 3 months before, SARS-CoV-2 diagnosis.	All patients on active treatment.	Same national dataset.	1:10; Age, sex^a^, comorbidity score, date of SARS-CoV-2 diagnosis.	Good
Nolan 2023	Cohort	Retrospective	Inpatient	7141	90,700	97,841	All Types	Any ICD-10 cancer diagnosis recorded during the index SARS-CoV-2 hospitalisation period.	NR	Same national health system database.	No matching.	Good
Kodde 2023	Cohort	Retrospective	Inpatient	1625	27,659	29,284	Lymphoma, Metastatic Cancers, Solid Cancers.	Presence of any of the three related Elixhauser comorbidities (lymphoma, metastatic cancer, solid tumor).	NR.	Same hospital cluster.	No matching.	Good
Udovica 2022	Cohort	Retrospective	Inpatient	89	156	245	All Types excluding Basal Cell Carcinoma	Cancer diagnosis/treatment within the last 6 months or metastatic/recurrent malignant disease.	Included patients on active treatment.	Same country, different hospital cluster.	No matching.	Poor
Bazgir 2022	Cohort	Retrospective	Inpatient	64	256	320	All Types	Cancer treatment in the last 2 months.	All patients on active treatment.	Same regional health data registry.	1:4; Age, gender.	Good
Plais 2022	Cohort	Prospective	Inpatient	105	315	420	All Types	Treatment/active surveillance within the last 6 months, or haematological cancer not in complete remission.	Included patients on active treatment.	Same hospital cluster.	1:3; Age, antimicrobial treatment, body mass index, comorbidities, hospitalisation within the last 3 months, immunosuppressive therapy, PaO2 at baseline, SAPS II and SOFA scores, sex, organ transplant.	Good
Chavez-MacGregor 2022	Cohort	Retrospective	Inpatient and outpatient	14,287	493,020	507,307	All Types	At least two ICD codes for cancer within 1 year before SARS-CoV-2 diagnosis.	Included patients on active treatment.	Same national electronic health record dataset.	1:5; Age, sex, comorbidities.	Good
Abuhelwa 2022	Cohort	Retrospective	Inpatient	27,760	1,022,285	1,050,045	Breast, colorectal, leukaemia, lung, lymphoma, multiple myeloma, prostate.	ICD-10 clinical modification codes of active cancer (C00–C99).	NR	From the same national health database.	No matching.	Good
Serraino 2021	Cohort	Retrospective	NR	466	38,268	38,734	All Types	<13 months since cancer diagnosis.	NR	Same regional health data registries.	No matching.	Good
Kim 2022	Cohort	Prospective	NR	10,426	253,179	263,605	All Types	Cancer diagnosed within 1-year before SARS-CoV-2 diagnosis.	Included patients on active treatment.	Same national electronic health record dataset.	Matched–NR; NR.	Good
Zhou 2023	Cohort	Retrospective	Inpatient and outpatient	142	5947	6089	All Types	Patients with pre-existing active malignancy who were not in complete remission.	NR	Same hospital cluster.	No matching.	Good
Raad 2023	Cohort	Retrospective	Inpatient and outpatient	1115	2851	3966	All Types	Cancer diagnosis/treatment within 1-year before SARS-CoV-2 diagnosis.	Included patients on active treatment.	Same multinational healthcare centres.	No matching.	Good
Rugge 2022	Cohort	Retrospective	Inpatient and outpatient	324	20,777	21,101	All Types	Cancer diagnosed within 1-year before SARS-CoV-2 diagnosis.	NR	Same regional health system data.	No matching.	Poor
Anantharaman 2021	Cohort	Retrospective	Inpatient and outpatient	33	4380	4413	All Types	Received cancer treatment in the 180 days prior to SARS-CoV-2 diagnosis.	All patients on active treatment.	Same health system registry.	No matching.	Poor
Johannesen 2021	Cohort	Retrospective	Inpatient and outpatient	53	7841	7894	All Types	<1 year since cancer diagnosis.	Included patients on active treatment.	Same epidemiological surveillance registry.	No matching.	Good
Sng 2020	Cohort	Retrospective	Inpatient	94	226	320	Solid cancers	Cancer diagnosis/treatment within the last 12 months or radiological/biochemical evidence of active/recurrent cancer.	Included patients on active treatment.	Same hospital cluster.	Matched–NR; Age, sex.	Poor
Alpert 2021	Cohort	Retrospective	Inpatient and outpatient	421	5135	5556	All Types	Newly diagnosed cancer or on active treatment.	Included patients on active treatment.	Same health system registry.	Matched–NR; Age, sex, comorbidities.	Poor
Bertuzzi 2021	Cohort	Retrospective	Inpatient	46	511	557	All Types	Presence of localised or metastatic disease at SARS-CoV-2 onset.	Included patients on active treatment.	Same hospital cluster.	1:4; Age, comorbidities.	Fair
Brar 2020	Cohort	Retrospective	Inpatient	117	468	585	All Types	Cancer treatment or active surveillance within 6 months of SARS-CoV-2 diagnosis and ongoing management.	Included patients on active treatment.	Same hospital cluster.	1:4; Age, sex, comorbidities.	Poor
Klein 2021	Cohort	Retrospective	Inpatient	77	324	401	All Types	Current haematological malignancy, metastatic disease, or cancer-directed systemic medical therapy within the last 6 months.	Included patients on active treatment.	Same hospital cluster.	1:2; Age, admission date, sex, race.	Poor
Dai 2020	Cohort	Retrospective	Inpatient	31	186	217	All Types	Active treatment within last 3 months.	All patients on active treatment.	Same hospital cluster.	1:6; Age, sex, region, time period.	Good

a All patients in Sullivan et al. 2023 were women.

The four main outcomes, mortality, ICU admission, severe SARS-CoV2 disease, and hospitalisation, were reported by 30 (100%), 22 (73·3%),^8^^,^^45^^,^^47^, ^48^, ^49^, ^50^^,^^52^, ^53^, ^54^, ^55^^,^^60^, ^61^, ^62^, ^63^, ^64^, ^65^, ^66^, ^67^^,^^69^, ^70^, ^71^^,^^73^ 15 (50·0%),^8^^,^^47^^,^^49^^,^^51^^,^^53^^,^^56^, ^57^, ^58^^,^^61^^,^^64^^,^^66^^,^^67^^,^^69^^,^^71^^,^^72^ and 12 (40·0%)^45^^,^^47^^,^^51^^,^^53^^,^^57^^,^^60^^,^^64^^,^^65^^,^^67^^,^^69^^,^^70^^,^^72^ of included studies, respectively. The definitions employed by each of the included studies for these outcomes are summarised in Table S7. Defined follow-up durations for mortality ranged from 28 days,^8^^,^^65^ to 3 months,^46^ following SARS-CoV-2 diagnosis. Of those providing definitions, two studies (6·7%) reported SARS-CoV-2-specific mortality,^51^^,^^54^ and 21 (70·0%) reported all-cause mortality.^8^^,^^45^, ^46^, ^47^^,^^49^^,^^52^^,^^53^^,^^55^, ^56^, ^57^^,^^59^, ^60^, ^61^, ^62^, ^63^, ^64^, ^65^, ^66^, ^67^, ^68^^,^^70^ ICU admission was infrequently defined and, in two cases (6·7%),^48^^,^^52^ was reported as a composite outcome alongside invasive ventilation and/or death.^8^^,^^52^^,^^74^^,^^75^ Definitions of severe disease requiring hospitalisation included guideline definitions from the National Health Commission in China,^61^ requirements for non-invasive ventilatory support,^8^^,^^47^^,^^53^^,^^54^^,^^56^, ^57^, ^58^^,^^64^^,^^69^^,^^72^ and clinical/biochemical characteristics of sepsis or acute respiratory distress syndrome.^49^^,^^66^ For one study (3·3%), severe disease was defined as composite outcomes including hospitalisation or death.^67^ Defined follow-up durations for hospitalisation ranged from 14^65^^,^^67^ to 45 days^64^ of SARS-CoV-2 diagnosis. Of the included 30 studies, five (16·7%) reported no demographic information for the cohort with active cancer (Table S8).^51^^,^^66^, ^67^, ^68^^,^^72^

Comorbidity details of included patients were reported by the majority of studies (*n* = 22/30; 73·3%).^8^^,^^18^^,^^46^, ^47^, ^48^, ^49^, ^50^^,^^52^^,^^54^, ^55^, ^56^, ^57^, ^58^, ^59^^,^^61^, ^62^, ^63^, ^64^, ^65^, ^66^^,^^70^^,^^71^ The comorbidities of patients with cancer reported most frequently by studies included diabetes (*n* = 20 studies), cardiovascular disease (*n* = 15), hypertension (*n* = 15), chronic kidney disease (*n* = 15 studies), and chronic lung disease (*n* = 16) (Table S9; Figure S3).

Most studies (*n* = 25, 96·2%) included all types of cancer except for Sng et al., 2020,^46^ Alsakarneh et al., 2024,^70^ and Sullivan et al., 2023,^69^ who included only solid cancers, colorectal cancers, and breast cancers, respectively. Information about cancer subtypes was reported by 13 studies (43·3%).^18^^,^^46^, ^47^, ^48^^,^^56^^,^^59^, ^60^, ^61^^,^^63^^,^^65^^,^^68^, ^69^, ^70^^,^^76^ The most common cancer type was lower gastrointestinal (*n* = 20,143) followed by urological (*n* = 11,144), breast (*n* = 9635), haematological *(n* = 8174*)* and thoracic *(n* = 7146*)* cancers. For 653 patients, the tumour type was recorded as ‘other’ (Table S10; Figure S4).

Six studies (20·0%) listed the anti-cancer therapies received by the cancer cohort.^46^^,^^50^^,^^67^^,^^69^, ^70^, ^71^ Chemotherapy (*n* = 72,39) was the most common therapy given (Table S11; Figure S5). Only three studies explicitly listed other anti-cancer therapies like hormonal therapy, immunotherapy, radiotherapy, surgery, and targeted therapy.^46^^,^^69^^,^^70^

For lineage imputation, 22,526,800 sequences were retrieved from NCBI GenBank via LAPIS and 20,343,148 sequences were retrieved from GISAID via Outbreak.info. There were limited differences between LAPIS and Outbreak queries, except in Singapore and Norway where no sequences were retrieved from LAPIS during the study period of Park et al., 2024,^72^ Raad et al., 2023,^57^ Hosseini-Moghaddam et al., 2023,^65^ and Johannesen et al., 2021^51^ which can be explained by the significantly higher number of sequences used for the Outbreak.info calculations (Figures S6 and S7). Global changes in VOC prevalence (Fig. 2) corroborated with previous trends.^77^

To factor for the intra-study variability of SARS-CoV-2 incidence across study periods and countries, we retrieved weekly new cases and new deaths of each country from the WHO COVID-19 database. In total, 647,943,787 new cases and 7,393,050 new deaths within the time periods and countries of the studies were considered (Figure S8). As the pandemic progressed, there was a decreasing trend in death per 1000 cases, reflecting improved treatment, immunity and non-pharmaceutical interventions. The inequality in sequencing performed and shared by different countries was reflected by the vast difference in LAPIS and Outbreak queried sequences per 1000 cases (Figure S8). The trend in number of sequences retrieved by LAPIS and Outbreak closely matches the trend of new cases and new deaths for each country-week, reflecting public health responses to increase sequencing capacity in response to pandemic waves (Figure S9). In the pre-Alpha emergence multinational study by Raad et al., 2023, peak incidence timings were different between countries, reflecting global epidemiology and differences in response (Figure S10).

The imputed dominant lineage for a majority of studies (19/30; 63·3%) were the pre-Alpha early clades (wild type, WT). We noted that Alpha was the dominant VOC in Udovica et al., 2022^54^; Delta was dominant in the WT to Delta period of Konermann et al. 2023^18^; Delta and Omicron reached a coalition majority in Leuva et al., 2022,^68^ Salvatore et al., 2023,^67^ and Turtle et al., 2023,^8^ and, as such, were placed into a separate Mixed Delta/Omicron category; and Omicron was imputed as the dominant variant in Starkey et al., 2023.,^45^ the Omicron study period of Konermann et al., 2023,^18^ Park et al., 2024,^72^ Li et al., 2024,^71^ and Alaskarneh et al., 2024.^70^

For the total cancer cohort, ten studies reported adjusted ORs of hospitalisation (573,193 cancer and 17,052,204 control),^45^^,^^47^^,^^51^^,^^53^^,^^60^^,^^64^^,^^67^^,^^69^^,^^70^^,^^72^ eleven studies reported adjusted ORs for severe SARS-CoV-2 infection (474,411 cancer and 2,355,246 control),^8^^,^^47^^,^^49^^,^^53^^,^^56^^,^^58^^,^^64^^,^^66^^,^^69^^,^^70^^,^^72^ thirteen studies reported adjusted ORs for ICU admission (190,446 cancer and 16,880,588 control),^8^^,^^45^^,^^47^^,^^49^^,^^53^^,^^60^^,^^63^^,^^64^^,^^66^^,^^67^^,^^69^, ^70^, ^71^ and nineteen studies reported adjusted ORs for mortality (626,977 cancer and 18,341,285 control).^8^^,^^45^^,^^47^^,^^49^^,^^53^^,^^54^^,^^56^, ^57^, ^58^^,^^60^^,^^63^^,^^64^^,^^66^, ^67^, ^68^, ^69^, ^70^, ^71^, ^72^ Meta-analysis of these adjusted ORs generated overall estimates of 1·58 (95% CI: 1·22–2·06) for hospitalisation, 1·00 (95% CI: 0·80–1·25) for severe SARS-CoV-2 infection requiring hospitalisation without ICU admission, 1·16 (95% CI: 0·94–1·42) for ICU admission, and 1·70 (95% CI: 1·36–2·12) for mortality (Figure S11a). The odds of mortality and hospitalisation, therefore, were significantly higher in patients with cancer than in patients without cancer. However, no such difference was detected with regards to the odds of ICU admission or severe SARS-CoV-2 infection. For the total cancer cohort, six studies, comprising 782 patients with cancer and 39,915 patients without cancer, reported adjusted HRs of mortality to yield an overall estimate of 1·86 (95% CI: 1·35–2·57 for the HR of mortality in patients with cancer.^46^^,^^50^^,^^52^^,^^55^^,^^61^^,^^62^ Conversely, the five studies that reported HRs of ICU admission generated an overall estimate for the HR of ICU admission of 2·24 (95% CI: 0·91–5·49) (Figure S11b).^48^^,^^50^^,^^52^^,^^61^^,^^62^

**Fig. 3:**
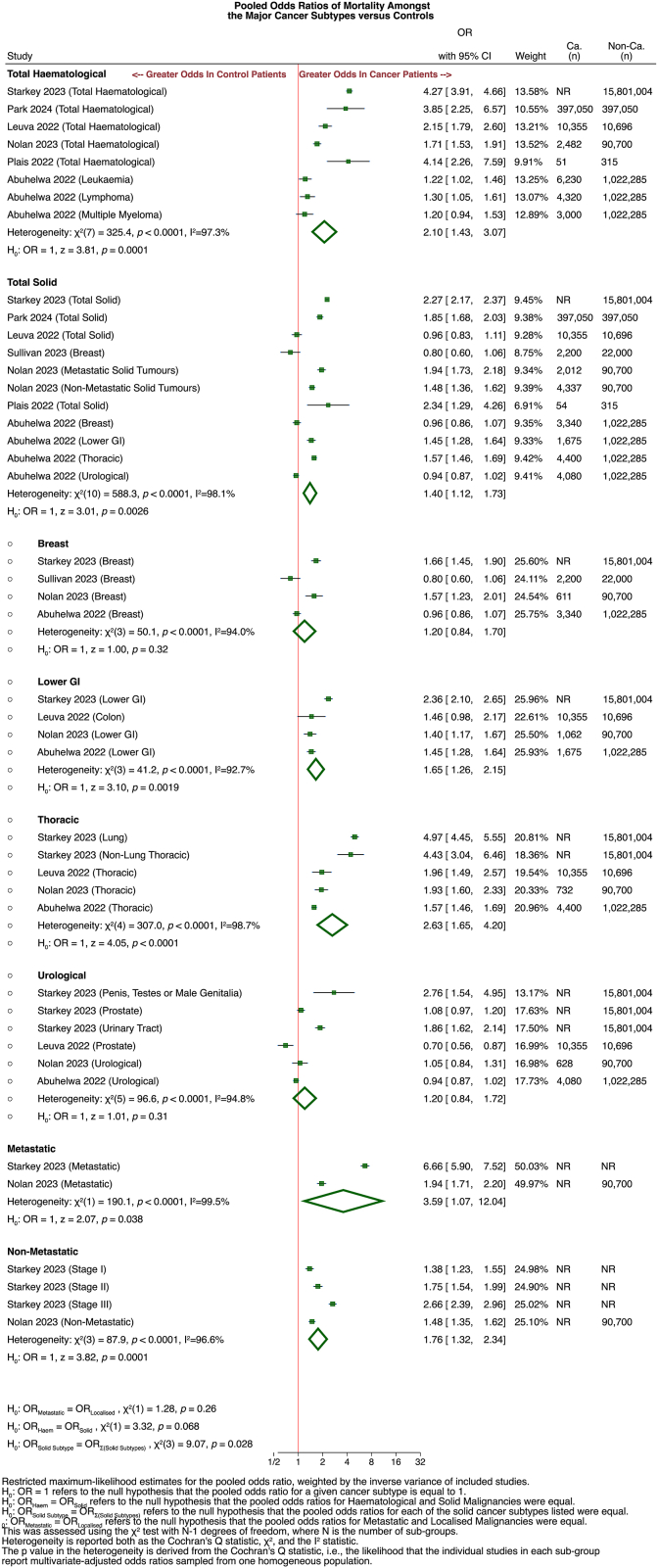
**Multivariate mortality odds ratio stratified by cancer types**. Multivariate mortality odds ratio of all cancers compared to controls without cancer stratified by cancer subtypes, including total haematological cancer, total solid cancer, solid cancer subtypes, and metastatic compared to non-metastatic outcomes.

The pooled OR of mortality in patients with solid tumours compared to controls without cancer was found to be 1·40 (95% CI: 1·12–1·73) from seven studies,^45^^,^^56^^,^^59^^,^^63^^,^^68^^,^^69^^,^^72^ while in patients with haematological malignancies this value was 2·10 (95% CI: 1·43–3·07). No significant difference between these two ORs was detected (Q (1) = 3·32; *p* = 0·068) (Fig. 3).

For the subset of patients known to have metastatic cancers, the overall mortality OR estimate was 3·59 (95% CI: 1·07–12·04), whilst the overall mortality OR estimate for patients known to have non-metastatic cancers was 1·76 (95% CI: 1·32–2·34), although only two studies were incorporated into either estimate. Of note, the ORs for mortality in patients with breast, thoracic, colorectal, or urological malignancy were calculated to be 1·20 (95% CI: 0·84–1·70), 2·63 (95% CI: 1·65–4·20), 1·65 (95% CI: 1·26–2·15) and 1·20 (95% CI: 0·84–1·72), respectively (Fig. 3).^78^ Although other solid malignancies were incorporated into the total cancer cohorts of many studies, insufficient studies provided adjusted ORs of mortality for the subgroups of patients with malignancies of the gynaecological, hepatocellular, musculoskeletal, or nervous systems to assess these through further subgroup analyses.

**Fig. 4:**
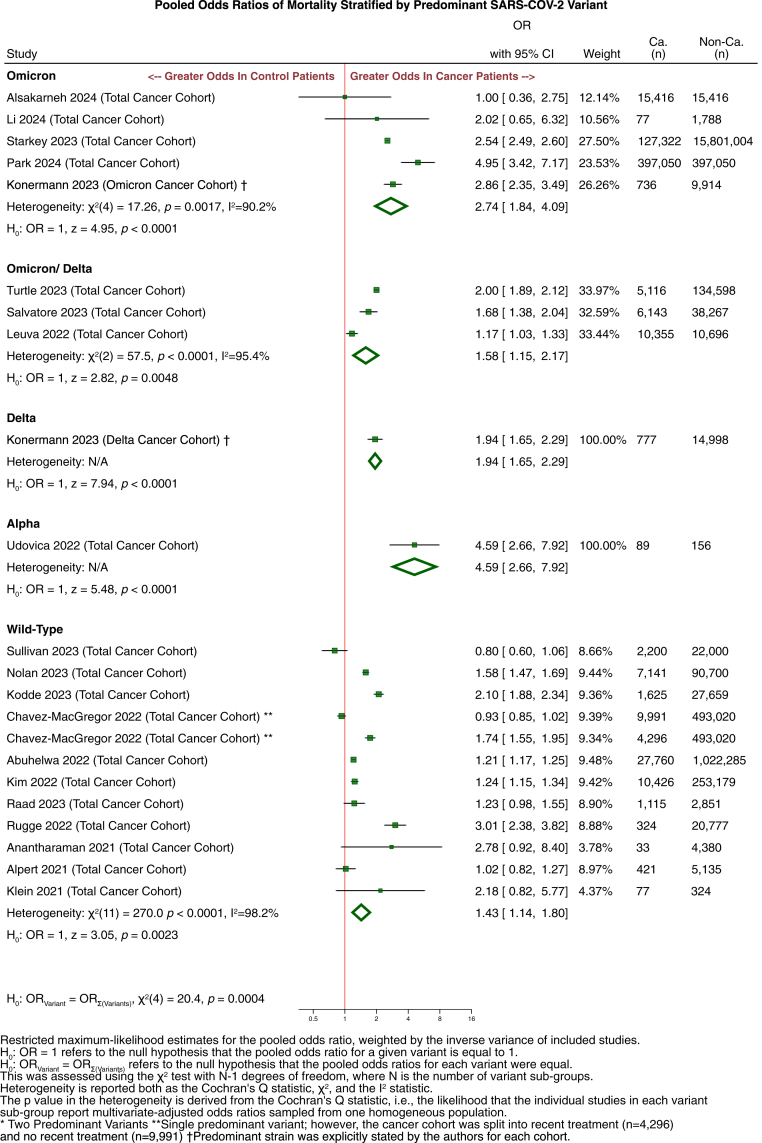
**Multivariate mortality odds ratio stratified by dominant variant**. Multivariate mortality odds ratio stratified by dominant variant of all cancers compared to controls without cancer, stratified by variant groups Omicron, Omicron/Delta, Delta, Alpha, and Wild-Type (Early Clades).

Additional subgroup analyses were conducted for the OR of mortality in patients with cancer, after stratification by the SARS-CoV-2 variant predominant in each study's geographical location during the study period. These yielded the following estimates for each strain: WT: 1·43 (95% CI: 1·14–1·80); Alpha: 4·59 (95% CI: 2·66–7·92); Delta: 1·94 (95% CI: 1·65–2·29); Mixed Delta/Omicron: 1·58 (95% CI: 1·15–2·17); Omicron: 2·74 (95% CI: 1·84–4·09). The OR of mortality in patients with cancer vs controls without cancer was found to differ significantly between these five categories of SARS-CoV-2 variants (Q (4) = 20·4; *p* = 0·0004) (Fig. 4).

The proportion of patients who had received ≥1 SARS-CoV-2 vaccination prior to their index case was reported in six studies.^8^^,^^45^^,^^63^^,^^66^, ^67^, ^68^ Subgroup analyses of these studies revealed no statistically significant difference (*p* = 0·77) in mortality ORs between the >50% majority vaccinated cohorts (OR: 1·73; 95% CI: 0·81–3·70), and <50% vaccinated cohorts (OR: 1·53; 95% CI: 1·13–2·07). For the ten study arms with ambiguous vaccination status,^18^^,^^47^^,^^49^^,^^53^^,^^54^^,^^56^, ^57^, ^58^^,^^60^^,^^64^ the OR of mortality was calculated to be 1·67 (95% CI: 1·32–2·29) (Figure S12).

Using the AHRQ standards, 21 studies (70·0%) were deemed to be of ‘good’ quality,^8^^,^^18^^,^^45^^,^^47^^,^^48^^,^^50^^,^^51^^,^^53^^,^^56^, ^57^, ^58^, ^59^^,^^61^, ^62^, ^63^^,^^65^^,^^68^, ^69^, ^70^, ^71^, ^72^ while one study (3·3%) was of ‘fair’ quality,^55^ and eight (26·7%) were of ‘poor’ quality (Table S1; Figure S13).^46^^,^^49^^,^^52^^,^^54^^,^^60^^,^^64^^,^^66^^,^^67^

**Fig. 5:**
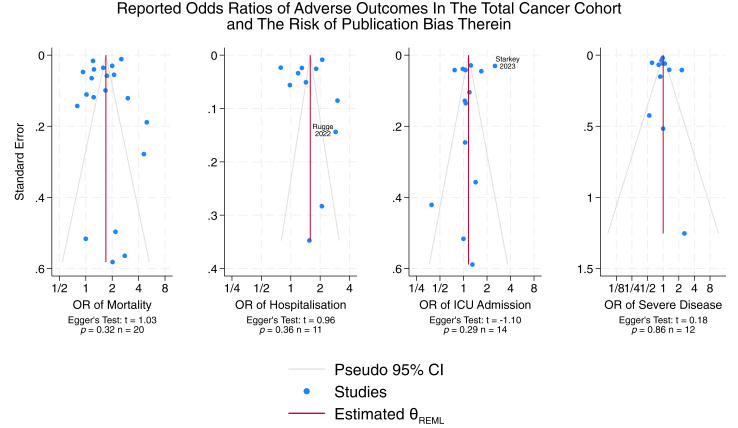
**Funnel Plot**. Quality assessment of mortality, hospitalisation, ICU admission, and severe disease in the total Cancer Cohort with COVID-19 to control patients via Funnel plot and Egger's test.

There was a significant degree of heterogeneity (Cochran's Q statistic *p* < 0·050) in each of the subgroups used to calculate ORs. For each of the four main outcomes, funnel plots exhibited a broadly symmetrical distribution of effect sizes across the included studies (Fig. 5), and thus, there was no discernible censorship of studies reporting any specific range of effect sizes. This interpretation should be valid for each outcome, as greater than 10 study arms were included in each.^78^ Funnel plots also identified several outliers, most notably the estimate of Starkey et al., 2023,^45^ for the OR of ICU admission, which may have been inflated due to their definition of ICU admission being a subset of patients that were hospitalised due to COVID-19. Egger's test statistic classified the estimates for all four outcomes as having a low risk of publication bias therein,^79^ although the validity of these conclusions is again limited due to the significant degree of heterogeneity in our data (Cochran's Q statistic *p* < 0·010 for each outcome).^78^

## Discussion

To our knowledge, this study is the largest meta-analysis to date evaluating outcomes in patients diagnosed with active cancer or treated for it within three years prior to their index SARS-CoV-2 case, thereby refining the risk assessment associated with cancer pathophysiology and treatment. We report a non-significant increase in mortality in patients with haematological malignancies compared to solid malignancies (both compared to controls without cancer), challenging assumptions that haematological cancers inherently carry a higher risk.^29^^,^^80^^,^^81^

This elevated risk in haematological cancers was previously demonstrated by large scale electronic healthcare record studies,^82^ and meta-analyses including Hardy et al. (2714 patients with haematological tumours vs 9343 patients with solid tumours—all-cause mortality OR = 1·64), and Khoury et al. (43,676 patients with cancer in total) showing a two-fold increase in case-fatality rate in patients with haematological cancers compared to patients with solid tumours.^4^^,^^81^ However, in contrast to our own analysis, these studies did not strictly filter for active cancer disease, for serological confirmation of SARS-CoV-2 infection, nor for multivariate analysis. Whilst our analysis also observed a higher OR for haematological malignancies, the difference was not statistically significant (*p* = 0·068), suggesting that the elevated risk may not be as pronounced when considering active cancer status and recent treatment history. Of the 16 studies included in Hardy et al. which reported greater odds of mortality in haematological cancers compared to solid tumours, only two met our stricter inclusion criteria (Table S6).^81^ Similarly, only one of the 14 studies incorporated into the analysis of haematological vs solid malignancies by Khoury and colleagues fulfilled our inclusion criteria (Table S4).^4^ Of note, all included studies in Hardy et al. and Khoury et al. were conducted prior to the rollout of vaccines and antiviral medications for the SARS-CoV-2 pandemic,^4^^,^^81^ whereas six of our studies explicitly reported vaccination status.^8^^,^^45^^,^^63^^,^^66^, ^67^, ^68^

Accordingly, their estimations of the OR of mortality are at high risk of being biased towards those studies assessing patients with non-active cancer. Indeed, a significant increase in the odds of mortality of patients with cancer on active treatment, compared to non-active treatment has previously been demonstrated in a UKCCMP cohort of 1044 patients with leukaemia; those on active treatment were found to have 125% greater odds of mortality, compared to patients with leukaemia diagnosed more than three years earlier.^13^ This supports the notion that by applying stricter case definition of active cancer or treatment within three years and using multivariate analysis to limit the impact of confounding factors, we have achieved a better resolution in the OR to which our most-vulnerable, immunocompromised population are exposed.

Analysis by Khoury and colleagues also demonstrated the importance of confounders, as mortality RR reduced from 112% to 69% when age and sex were matched.^4^ In our analysis which exclusively selects for studies with multivariate adjusting for age, sex, and other factors such as comorbidities, vaccination status, and socioeconomic status, we observed further reduced mortality odds. Our results were confirmed by concordant statistical analysis across two platforms (Stata and Python).

We have included 281,270 patients with active cancer in our study, in which all are compared to healthy controls in a multivariate manner, as earlier studies have shown that age, sex, and comorbidity status are significant risk factors.^4^^,^^83^ Nonetheless, there is still significant heterogeneity in our included studies. In order to limit confounding in our meta-analysis, we excluded any ORs or HRs not weighted by cohort-level covariates, i.e., univariate parameters. Nonetheless, this approach would not have eliminated all potential confounding in our restricted maximum-likelihood estimates, since each included study utilised a different constellation of covariates against which to weight their final multivariate estimates (Table S7). Uncertainty is further compounded by heterogeneity in the definition of active cancer used by different studies, with a gap from 2 months to 3 years between cancer diagnosis/most recent cancer treatment and index SARS-CoV-2 case (Table 1). However, this is still likely to have improved the accuracy of our final estimates through eliminating the confounding effects attributed by these covariates in the individual studies which considered them. The different mortality OR between variant waves suggest that dominant variants, as well as the changes in community immune status, COVID-19 treatment, and non-pharmaceutical interventions (NPIs) are contributing factors towards heterogeneity.

While none of our included studies presented SARS-CoV-2 sequencing data or reported SGTF which can be used to identify Alpha and Omicron variants,^17^ we demonstrate that lineage imputation can be used to verify claims in variant identification. The imputation of 45–50% pre-Alpha early WT clades in the designated Alpha period (Wave 2) in Turtle et al., 2023,^8^ highlights the importance of pathogen sequencing, as studies may use international, epidemiological, or study-defined cut-offs rather than strict genomic definitions. For genomic imputation to be reliable, it requires: 1) circulation of only a single variant in the defined study period and locations, and 2) sufficient sequencing that is publicly available, which is currently challenged by global disparity in resources and sequence-sharing practices.^77^^,^^84^ The sequential emergence of COVID-19 variants and study periods spanning multiple variants,^8^^,^^45^^,^^58^^,^^67^^,^^68^ made it difficult to confidently attribute dominant lineage. We encourage the future sharing of pathogen genome or lineage data in future infectious disease studies and expect that genomic imputation will become a widespread technique in the future studies. Where studies span across transitions between VOCs, genomic imputation is less accurate without further details into the temporal and geographical distribution of admissions and disease progression. This can be compounded by the lagged introduction of cases in multinational studies, as demonstrated by Raad 2023,^57^ and by the potential differences in the vulnerability and health-seeking behaviour between patients with cancer and patients without cancer. Of all 30 included studies, only Raad 2023 was a multinational study. As we did not have data regarding the patient distribution across countries in this study, we estimated that the case distribution was equal across countries. Although this may have been an inaccurate estimate, it did not affect the imputation as a supermajority of the SARS-CoV-2 variants in Raad 2023 were WT/Early-clade. Thus, for multicentre studies, we recommend the reporting of patient composition from each study centre.

Interestingly, our results reveal a higher relative mortality risk for patients with cancer during the SARS-CoV-2 Alpha and Omicron variant waves compared to the WT and Delta waves. This diverges from the observed patterns in the general population where there is an increase in mortality from WT to Alpha, and then a decrease in disease mortality in Delta and Omicron.^85^ This suggests a nuanced virus–host interaction or difference in vaccine response in patients with cancer that may not correlate with broader epidemiological trends in patients without cancer. Our finding is supported by a CDC cross-sectional analysis which showed a 38% increase in mortality in the SARS-CoV-2 lymphoma group, in stark contrast to a 21% decrease in the general population when comparing the Omicron wave to the WT wave.^86^ This may be more broadly applicable to patients who are immunocompromised, as a similar increase in relative mortality risk from Delta to Omicron is observed in a cohort study comparing people living with HIV to people without HIV.^87^ Thus, although overall mortality risk decreases for both patients with cancer and patients without cancer as the pandemic progressed and vaccines rolled out, patients with cancer benefit less than patients without cancer.^3^

Our analyses demonstrated no significant difference in the odds or the hazards of ICU admission, as well as the odds of severe COVID-19 infection, in patients with cancer compared to patients without cancer. Conversely, there were significantly greater odds of hospital admission in the former cohort, which may represent an innate bias of acute medicine physicians to admit patients with cancer over patients without cancer. Our analyses also support this bias, given the significant increase in the odds of mortality in patients with cancer and in their mortality per unit time compared with controls without cancer.

This study has a number of limitations, with the majority arising from a lack of consensus in the available literature including: 1) a need for more granular data on the timing of cancer diagnosis, cancer treatments (and their specifics) relative to SARS-CoV-2 infection; 2) a lack of consensus definitions between studies (for example criteria for severe disease without ICU admission, or criteria for ICU admission) (Table S7); 3) limitations due to the types of studies available: 5/30 studies (16·7%) were prospective, whilst the rest (25/30; 83·3%) were retrospective studies that leveraged electronic health records; 4) variability in the follow-up periods recorded (ranging between 30 and 90 days); 5) variability in patient matching which was employed in only 15/30 (50·0%) of included studies,^46^^,^^47^^,^^49^^,^^50^^,^^52^^,^^53^^,^^55^^,^^59^^,^^61^^,^^66^^,^^68^, ^69^, ^70^, ^71^, ^72^ of which only ten included comorbidity index matching^49^^,^^52^^,^^53^^,^^55^^,^^59^^,^^68^, ^69^, ^70^, ^71^, ^72^ (we mitigated this factor by only including multivariate analysis data); 6) the effect of including studies from the start of the COVID-19 pandemic (when anti-viral therapies and steroids were not routinely used) in addition to studies later on in subsequent waves (when such treatments and vaccination was available); 7) limited vaccination status reporting in studies undertaken after vaccination become prevalent, as well as measurement of immune responses following vaccine in the setting of immune compromise; and 8) a lack of SARS-CoV-2 sequencing data available for any of the included studies. Furthermore, this meta-analysis was limited by low numbers of studies reporting data on recent anti-cancer treatment regimens, especially immunotherapies and targeted therapeutic agents, compounded by our specific focus comparing to controls without cancer. In addition, there were limited studies that compared patients with cancer on active cancer therapy compared to controls without cancer, as the impact of anti-cancer treatment modalities is more naturally addressed by comparing outcomes in patients with cancer who are actively treated compared to those who are not actively treated. We echo the findings of Khoury et al. in their earlier-meta-analysis,^4^ that future studies need to report patient-level data on type and recency of anti-cancer therapies received for future work to interrogate the clinical significance of patients with cancer receiving different treatment modalities when diagnosed with COVID-19. Additional limitations include the impact of hospital admission biases towards patients with cancer on the interpretation of outcomes, as well as the limitations imposed by the data biases towards Western European countries and North America, which may not be representative of the global cancer population.

This large, comprehensive systematic review and meta-analysis confirms previous findings that patients with cancer have a poorer SARS-CoV-2 mortality than patients without cancer. Furthermore, our results report no significant difference in mortality between patients with solid and haematological malignancies when compared to controls without cancer, challenging assumptions that haematological malignancies inherently carry a higher risk. Last, our results reveal a higher mortality risk for patients with cancer during the SARS-CoV-2 Alpha and Omicron variant waves, diverging from the observed VOC-associated mortality patterns in the general population, suggesting differential host-dependent pathogenicity mechanisms. Thus, it is necessary to consider the impact on vulnerable populations when responding to endemic outbreaks. Future infectious disease outcome studies including patients with cancer should clearly define the recency of cancer diagnosis and treatment(s) in relation to the index infection, as well as integrate vaccination status and VOC-sequencing to enable better risk stratification and guide clinical decision-making.

## Contributors

MTKC, DMF, and RKG were responsible for conceptualisation of the study. MTKC, JSM, SFHS, AT, JCX, NS, LHF, AI, JTNC, SS, MKH, AB, AP, GDM, MH, AOF, and CCY participated in screening, data extraction and quality assessment, with supervision by DMF. Genomic imputation was performed by MTKC. Statistical analyses were conducted by JSM and reviewed by ES. MTKC and DMF wrote the manuscript. All authors provided editorial input. MTKC, DMF, JSM, SFHS, and AT have access to and verified the underlying data.

## Data sharing statement

The raw data and code used in this project can be found at https://github.com/TKMarkCheng/scerc2.

## Editor note

The *Lancet* Group takes a neutral position with respect to territorial claims in published maps and institutional affiliations.

## Declaration of interests

All authors declare no competing interests.
