# Supplementary material for: Determinants of SARS-CoV-2 outcomes in patients with cancer vs controls without cancer: a multivariable meta-analysis with genomic imputation
Source: eClinicalMedicine. 2025 May 2;83:103194. doi: 10.1016/j.eclinm.2025.103194 (PMC12123352; doi:10.1016/j.eclinm.2025.103194)

SUPPLEMENTARY MATERIAL

Table of Contents

Document S1: Detailed search strategies 2

Document S2: Detailed inclusion and exclusion criteria 6

Table S1: Newcastle-Ottawa Scale quality assessment outcomes 8

Table S2: Clade groupings 9

Table S3: Geographical distribution of cancer patients across 30 included studies 10

Table S4: Comparison of studies included in this review versus studies included in Khoury et al. 2022 11

Table S5: Comparison of studies included in this review versus studies included in Felice et al. 2022 14

Table S6: Comparison of studies included in this review versus studies included in Han et al. 2022 18

Table S7: Outcome definitions within 30 included studies 20

Table S8: Comparison of demographic characteristics in cancer vs non-cancer patients in the 30 included studies 26

Table S9: Comparison of comorbidity status in cancer vs non-cancer patients in the 30 included studies 28

Table S10: Breakdown of cancer subtypes across 30 included studies 30

Table S11: Breakdown of cancer treatments across the 30 included studies 32

Supplementary Figure 1 - Geographical distribution of cancer patients across 30 studies 33

Supplementary Figure 2 - Geographical distribution of the 30 included studies 34

Supplementary Figure 3 - Heatmap showing percentage of comorbidities in included studies' cancer and control cohorts 35

Supplementary Figure 4 - Number of patients and number of studies by cancer types 36

Supplementary Figure 5 - Number of patients and studies which describe different forms of anti-cancer therapy for studies which provided a breakdown 37

Supplementary Figure 6 - Total number of sequences queried, stratified by study 38

Supplementary Figure 7 - Total number of sequences queried, stratified by country 39

Supplementary Figure 8 - Total number of new SARS-CoV-2 cases and deaths reported to WHO, and number of sequences analysed, stratified by study period and country 40

Supplementary Figure 9 - the weekly evolution of major variants sequenced, and epidemiological trends of new cases and new deaths reported to WHO in the Multinational study Raad 2023 41

Supplementary Figure 10 - the weekly evolution of major variants sequenced, and epidemiological trends of new cases and new deaths reported to WHO in studies which reported dominance of a VOC 42

Supplementary Figure 11a - Pooled odds ratios of hospitalisation, ICU admission, mortality, and severe disease in COVID-19 patients with any type of cancers compared with those without cancer 43

Supplementary Figure 11b - Pooled hazard ratios of ICU admission and mortality in COVID-19 patients with any type of cancers compared with those without cancer 44

Supplementary Figure 12 - Pooled odds ratio of mortality in cancer patients compared to non-cancer patients of different vaccination status 45

Supplementary Figure 13 - AHRQ scores of study quality based on Newcastle-Ottawa Quality Assessment Scale assessment 46

Document S1: Detailed search strategies

A comprehensive search of electronic databases including Medline, Embase, Cochrane Central Register of Controlled Trials (CENTRAL), Cochrane Database of Systematic Reviews, and the World Health Organization COVID-19 database were conducted for publications between 1 Jan 2019 and 22 Nov 2024.

The search results are laid out below.

Database: Medline (Ovid MEDLINE® and Epub Ahead of Print, In-Process, In-Data-Review & Other Non-Indexed Citations, Daily and Versions) [1946 to November 22, 2024]

1	exp Severe Acute Respiratory Syndrome/ or exp SARS-CoV-2/ or exp Severe acute respiratory syndrome-related coronavirus/	189815
2	exp COVID-19/	281193
3	(corona* adj1 (virus* or viral*)).ti,ab,kw,kf.	6999
4	(CoV not (Coefficien* or "co-efficien*" or covalent* or Covington* or covariant* or covarianc* or "cut-off value*" or "cutoff value*" or "cut-off volume*" or "cutoff volume*" or "combined optimi?ation value*" or "central vessel trunk*" or CoVR or CoVS)).ti,ab,kw,kf.	148746
5	(coronavirus* or 2019nCoV* or 19nCoV* or "2019 novel*" or Ncov* or "n-cov" or "SARSCoV-2*" or "SARSCoV-2*" or SARSCoV2* or "SARS-CoV2*" or "severe acute respiratory syndrome*" or COVID*2).ti,ab,kw,kf.	438813
6	1 or 2 or 3 or 4 or 5	469727
7	exp Neoplasms/	4044667
8	exp Immunotherapy/ or exp Immunotherapy, Active/	353886
9	exp Radiotherapy/	214690
10	("Cancer*" or "malignan*" or "carcinoma*" or "myeloma*" or "leuk?emia" or "tumo?r" or "nephroblastoma*" or "blastoma*" or "immunotherapy" or "chemotherapy" or "neoplasm*" or "oncolog*" or "radiotherapy").ti,ab,kw,kf.	4537335
11	7 or 8 or 9 or 10	5777083
12	exp Hospital Mortality/ or exp Mortality/	434649
13	exp Intensive Care Units/	113208
14	exp Critical Illness/ or Critical Care/	97052
15	exp "Severity of Illness Index"/	289258
16	exp Patient Admission/ or exp Hospitalization/	309141
17	exp Intubation/ or exp Intubation, Intratracheal/	59455

18	exp Noninvasive Ventilation/	4050
19	("mortality" or "fatality" or "death*" or "severity" or "hospitali*" or "high dependency" or "intensive care" or "critical care" or "critical* ill*" or "intensive therapy" or "ITU" or "ICU" or "CCU" or "oxygen" or "ventilat*" or "intubat*").ti,ab,kw,kf.	3621180
20	12 or 13 or 14 or 15 or 16 or 17 or 18 or 19	4155316
21	6 and 11 and 20	12794
22	21 not (letter or historical article or comment or editorial or news or conference abstract status).pt. not (Animals/ not humans/)	12148
23	limit 22 to yr="2019-Current"	12015

Database: Embase [1974 to November 22, 2024]

1	severe acute respiratory syndrome coronavirus 2/ or coronavirus disease 2019/ or experimental coronavirus disease 2019/	452142
2	(corona* adj1 (virus* or viral*)).ti,ab.	5763
3	(CoV not (Coefficien* or co-efficien* or covalent* or covington or covariant* or covarianc* or "cut-off value*" or "cutoff value*" or "cut-off volume*" or "cutoff volume*" or "combined optimi?ation value*" or "central vessel trunk" or CoVR or CoVS)).ti,ab.	154951
4	(coronavirus* or 2019nCoV* or 19nCoV* or "2019 novel*" or Ncov* or "n-cov" or "SARSCoV-2*" or "SARSCoV-2*" or SARSCoV2* or "SARS-CoV2*" or "severe acute respiratory syndrome*" or COVID*2).ti,ab.	496423
5	1 or 2 or 3 or 4	569082
6	*cancer/ or *malignant neoplasm/ or *neoplasm/	255802
7	*active immunotherapy/ or *cancer immunotherapy/ or *immunotherapy/	94417
8	*cancer radiotherapy/ or *radiotherapy/	120672
9	("Cancer*" or "malignan*" or "carcinoma*" or "myeloma*" or "leuk?emia" or "tumo?r" or "nephroblastoma*" or "blastoma*" or "immunotherapy" or "chemotherapy" or "neoplasm*" or "oncolog*" or "radiotherapy").ti,ab.	6036263
10	6 or 7 or 8 or 9	6087092
11	*hospital mortality/ or *cancer mortality/ or *all cause mortality/ or *in-hospital mortality/ or *mortality/	162227
12	*intensive care unit/ or *intensive care/ or *critically ill patient/ or *high dependency unit/ or *critical illness/	148746
13	*Pneumonia Severity Index/ or *COVID-19 Severity score/ or *quick COVID-19 severity index/	177
14	*hospital admission/ or *hospitalization/	76070
15	*endotracheal intubation/ or *intubation/	24795
16	*noninvasive ventilation/	6127

17	("mortality" or "fatality" or "death*" or "severity" or "hospitali*" or "high dependency" or "intensive care" or "critical care" or "critical* ill*" or "intensive therapy" or "ITU" or "ICU" or "CCU" or "oxygen" or "ventilat*" or "intubat*").ti,ab.	5057869
18	11 or 12 or 13 or 14 or 15 or 16 or 17	5114119
19	5 and 10 and 18	14312
20	19 not (letter or editorial).pt. not (nonhuman/ not human/) not (conference abstract or conference paper or conference proceeding or "conference review").pt.	7956
21	limit 20 to yr="2019-current"	7886

Database: Cochrane (CCRCT, CDSR) [November 22, 2024]

#1	MeSH descriptor: [SARS-CoV-2] explode all trees	3544
#2	MeSH descriptor: [COVID-19] explode all trees	8321
#3	(coronavirus*) or (2019nCoV*) or (19nCoV*) or (2019 novel*) or (SARS-CoV2*) or (severe acute respiratory syndrome*)	28060
#4	#1 OR #2 OR #3	31627
#5	MeSH descriptor: [Neoplasms] explode all trees	127536
#6	MeSH descriptor: [Leukemia] explode all trees	6670
#7	MeSH descriptor: [Immunotherapy] explode all trees	12335
#8	MeSH descriptor: [Radiotherapy] explode all trees	10167
#9	MeSH descriptor: [Drug Therapy] explode all trees	186928
#10	(Cancer*) OR (malignan*) OR (carcinoma*) OR (myeloma*) OR (leuk*mia) OR (tumo*r) or (nephroblastoma*)OR (*blastoma*) OR (immunotherapy) OR (chemotherapy) OR (neoplasm*) OR (oncolog*) OR (radiotherapy)	336195
#11	#5 OR #6 OR #7 OR #8 OR #10	349770
#12	MeSH descriptor: [Mortality] explode all trees	18997
#13	MeSH descriptor: [Patient Acuity] explode all trees	27191
#14	MeSH descriptor: [Intensive Care Units] explode all trees	6247
#15	MeSH descriptor: [Critical Illness] explode all trees	3783
#16	MeSH descriptor: [Critical Care] explode all trees	3117
#17	(mortality) or (fatality) or (death*) or (severity) or (hospitali*) or (high dependency) or (intensive care) or (critical care) or (critical* NEXT ill*) or (intensive therapy) or (ITU) or (ICU) or (CCU) or (oxygen) or (ventilat*) or (intubat*)	497368
#18	#12 OR #13 OR #14 OR #15 OR #16 OR #17	500439

#19	#4 AND #11 AND #18 with Cochrane Library publication date from Jan 2019 to present	2484
	Cochrane Central Register of Controlled Trials	2056
	Cochrane Database of Systematic Reviews	353

Database: WHO COVID-19 Research Database [October 15, 2023] (content spanned the time period March 2020 to June 2023, no longer searchable since January 2024)

(kw:("Cancer*" OR "malignan*" OR "carcinoma*" OR "myeloma*" OR "leuk?emia" OR "tumo?r" OR "nephroblastoma*" OR "blastoma*" OR "immunotherapy" OR "chemotherapy" OR "neoplasm*" OR "oncolog*" OR "radiotherapy") OR tw:("Cancer*" OR "malignan*" OR "carcinoma*" OR "myeloma*" OR "leuk?emia" OR "tumo?r" OR "nephroblastoma*" OR "blastoma*" OR "immunotherapy" OR "chemotherapy" OR "neoplasm*" OR "oncolog*" OR "radiotherapy"))

AND

(kw:("mortality" OR "fatality" OR "death*" OR "severity" OR "hospitali*" OR "high dependency" OR "intensive care" OR "critical care" OR "critical* ill*" OR "intensive therapy" OR "ITU" OR "ICU" OR "CCU" OR "oxygen" OR "ventilat*" OR "intubat*") OR tw:("mortality" OR "fatality" OR "death*" OR "severity" OR "hospitali*" OR "high dependency" OR "intensive care" OR "critical care" OR "critical* ill*" OR "intensive therapy" OR "ITU" OR "ICU" OR "CCU" OR "oxygen" OR "ventilat*" OR "intubat*"))

AND

entry date:([20191001 TO 20231016])

Results: 15,059

Document S2: Detailed inclusion and exclusion criteria

	Include	Exclude
**Population**	• All age, all gender<br>• Patients with reverse transcription polymerase chain reaction (RT-PCR) or rapid antigen test confirmed SARS-CoV-2 infection.<br>• 10 or more patients with SARS-CoV-2 AND exposure (active cancer) described.	• Less than 10 patients with SARS-CoV-2 AND exposure (active cancer) described.
**Exposure**	• Patients with active cancer (defined as cancer diagnosis or cancer treatment within three years of index SARS-CoV-2 infection).	• Less than 10 patients with SARS-CoV-2 AND exposure (active cancer) described.
**Comparator/ Context**	• Studies that compare active cancer vs non-cancer.<br>• At Title and Abstract screening, include papers which only describe cancer as some papers only describe control patients in full text.	• Studies that only describe a cancer cohort without a non-cancer control.
**Outcome**	• Comparing one or more of all-cause/COVID-19 specific mortality, ICU admission/intubation incidence, SARS-CoV-2 infection severity, and hospital admission relative to all SARS-CoV-2 infection cases (i.e. active cancer vs. non-cancer controls) using multivariable statistics.<br>• At Title and Abstract screening, include papers which only describe cancer as some papers only describe control patients in full text.	• Only morbidity and/or mortality of Cancer or COVID-19 is described.
**Study Characteristics**	• Peer-reviewed observational studies, cohort, case-control.<br>• Meta-analyses with NEW, previously unpublished data.	• Narrative reviews, conference abstracts, correspondences, perspectives, opinion articles.<br>• Case reports and case series.<br>• Non-human studies (e.g. animal experiments, in vitro observations).
**Others**	• Patients confirmed with SARS-CoV-2 infection via RT-PCR positive test or rapid antigen tests. This includes patients given ICD-10 codes for laboratory (as opposed to unconfirmed) SARS-CoV-2 diagnosis (which is U07.1).<br>• Grey (preprints, non-peer-reviewed) literature.<br>• English OR Chinese OR Spanish.<br>• Patients confirmed with SARS-CoV-2 infection via RT-PCR positive test or rapid antigen tests. This includes patients given ICD-10 codes for laboratory (as opposed to unconfirmed) SARS-CoV-2 diagnosis (which is U07.1).	• Patients not confirmed with RT-PCR or rapid antigen test.<br>• The full paper is not available (Please consult other team members before excluding solely on this criteria)

Table S1: Newcastle-Ottawa Scale quality assessment outcomes

Study	Cohort selection score	Cohort comparability score	Cohort outcome score	Total	AHRQ score
Alsakarneh 2024	4	2	3	9	Good
Li 2024	4	2	3	9	Good
Starkey 2023	3	2	2	7	Good
Konermann 2023	3	2	3	8	Good
Park 2024	4	2	2	8	Good
Turtle 2023	3	2	2	7	Good
Salvatore 2023	2	2	1	5	Poor
Leuva 2022	4	2	2	8	Good
Hosseini-Moghaddam 2023	3	2	2	7	Good
Sullivan 2023	3	2	3	8	Good
Nolan 2023	3	2	2	7	Good
Kodde 2023	3	2	2	7	Good
Udovica 2022	1	2	2	5	Poor
Bazgir 2022	3	2	3	8	Good
Plais 2022	3	2	3	8	Good
Chavez-MacGregor 2022	3	2	3	8	Good
Abuhelwa 2022	3	2	3	8	Good
Serraino 2021	3	2	3	8	Good
Kim 2022	4	2	3	9	Good
Zhou 2023	3	2	3	8	Good
Raad 2023	3	2	3	8	Good
Rugge 2022	3	2	1	6	Poor
Anantharaman 2021	3	2	1	6	Poor
Johannesen 2021	4	2	2	8	Good
Sng 2020	2	2	1	5	Poor
Alpert 2021	3	2	1	6	Poor
Bertuzzi 2021	2	1	2	5	Fair
Brar 2020	3	2	1	6	Poor
Klein 2021	3	1	1	5	Poor
Dai 2020	3	2	2	7	Good

AHRQ, Agency for Healthcare Research and Quality.

Table S2: Clade groupings

Nextstrain Clade	Common Name	Colour	Parent	WHO
19A		#f1f6fa		Early-Clade/WT
19B		#e6ecf0	19A	Early-Clade/WT
20A		#dbdfe6	19A	Early-Clade/WT
20B		#bec0ca	20A	Early-Clade/WT
20C		#d1d5dd	20A	Early-Clade/WT
20D		#b4b6c0	20B	Early-Clade/WT
20E		#a3a3a3	20A	Early-Clade/WT
20F		#9b9b9b	20B	Early-Clade/WT
20G		#929292	20C	Early-Clade/WT
20H	Beta	#6c00c1	20C	Beta
20I	Alpha	#5e00d9	20B	Alpha
20J	Gamma	#5b1aee	20B	Gamma
21A	Delta	#6148f8	20A	Delta
21B	Kappa	#6270d8	20A	Kappa
21C	Epsilon	#6a84d5	20C	Epsilon
21D	Eta	#80a3f5	20A	Eta
21E	Theta	#78a1c3	20B	Theta
21F	Iota	#7facb7	20C	Iota
21G	Lambda	#86b7ab	20D	Lambda
21H	Mu	#8dbe9d	20A	Mu
21I	Delta	#5455cb	21A	Delta
21J	Delta	#758af9	21A	Delta
21K	BA.1	#8bbae9	21M	Omicron
21L	BA.2	#95cad7	21M	Omicron
21M	Omicron,B.1.1.628	#a1b5af	20B	Omicron
22A	BA.4	#a9e3b0	21L	Omicron
22B	BA.5	#b4e99f	21L	Omicron
22C	BA.2.12.1	#c0ef8f	21L	Omicron
22D	BA.2.75	#ccf282	21L	Omicron
22E	BQ.1	#daf578	22B	Omicron
22F	XBB	#e6f370	21L	Omicron
23A	XBB.1.5	#f1ee6a	22F	Omicron
23B	XBB.1.16	#f4e865	22F	Omicron
23C	CH.1.1	#f1dc60	22D	Omicron
23D	XBB.1.9	#edcb5b	22F	Omicron
23E	XBB.2.3	#e8b555	22F	Omicron
23F	EG.5.1	#e49b4e	23D	Omicron
23G	XBB.1.5.70	#df7b46	23A	Omicron
23H	HK.3	#dc5c40	23F	Omicron
23I	BA.2.86	#da3f3a	21L	Omicron

Based on: https://nextstrain.org/ncov/gisaid/global/all-time.

Table S3: Geographical distribution of cancer patients across 30 included studies

Country	Number of patients with cancer and SARS-CoV-2	Number of control patients with SARS-CoV-2	Number of studies
USA	94376	1955870	13
UK	132532	15935828	3
China	250	7921	3
Italy	836	59556	3
Germany	3138	52571	2
France*	105	315	2
South Korea*	40334	397050	2
Norway	53	7841	1
Iran	64	256	1
Canada	8378	456196	1
Austria	89	156	1
Australia*	NR	NR	1
Brazil*	NR	NR	1
Japan*	NR	NR	1
Lebanon*	NR	NR	1
Singapore*	NR	NR	1
Spain*	NR	NR	1

*It was not possible to include patient numbers from Raad et al. 2023 as it was a multinational study that did not stratify cancer patients by country of origin.

Table S4: Comparison of studies included in this review versus studies included in Khoury et al. 2022

Khoury et al. Reference number	Khoury et al. reference name (n=19)	Papers in our study (n=30)	Definition of cancer	Reason for exclusion	Inclusion status
		Alsakarneh 2024	ICD-10 clinical modification codes of colorectal cancer (C18-C20).		exclusive to us
		Li 2024	Active cancer or those diagnosed with cancer within 1 year of developing community-acquired COVID-19 pneumonia.		exclusive to us
		Starkey 2023	Patients in a national cancer registry.		exclusive to us
		Konermann 2023	Active cancer based on relavant ICD codes.		exclusive to us
		Park 2024	First ICD-10 cancer diagnosis code (C00–99) <1 year before SARS-CoV-2 diagnosis.		exclusive to us
		Turtle 2023	Active cancer diagnosis and treatment.		exclusive to us
		Salvatore 2023	Most recent cancer diagnosis within the last 3 years.		exclusive to us
		Leuva 2022	Active diagnosis of new or previously established cancer in the last 2 years.		exclusive to us
		Hosseini-Moghaddam 2023	Cancer treatment in the last 6 months or diagnosis within the last 1-year.		exclusive to us
		Sullivan 2023	At least two ICD codes for cancer within 1 year before, and anticancer treatment within 3 months before, SARS-CoV-2 diagnosis.		exclusive to us
		Nolan 2023	Any ICD-10 cancer diagnosis recorded during the index SARS-CoV-2 hospitalisation period.		exclusive to us
		Kodde 2023	Presence of any of the three related Elixhauser comorbidities (lymphoma, metastatic cancer, solid tumor).		exclusive to us
		Udovica 2022	Cancer diagnosis/ treatment within the last 6 months or metastatic/ recurrent malignant disease.		exclusive to us
		Bazgir 2022	Cancer treatment in the last 2 months.		exclusive to us
		Plais 2022	Treatment/active surveillance within the last 6 months, or haematological cancer not in complete remission.		exclusive to us
		Chavez-MacGregor 2022	At least two ICD codes for cancer within 1 year before SARS-CoV-2 diagnosis.		exclusive to us
		Abuhelwa 2022	ICD-10 clinical modification codes of active cancer (C00-C99).		exclusive to us
		Serraino 2021	<13 months since cancer diagnosis.		exclusive to us
		Kim 2022	Cancer diagnosed within 1-year before SARS-CoV-2 diagnosis.		exclusive to us
		Zhou 2023	Patients with pre-existing active malignancy who were not in complete remission.		exclusive to us
		Raad 2023	Cancer diagnosis/ treatment within 1-year before SARS-CoV-2 diagnosis.		exclusive to us
		Rugge 2022	Cancer diagnosed within 1-year before SARS-CoV-2 diagnosis.		exclusive to us
		Anantharaman 2021	Received cancer treatment in the 180 days prior to SARS-CoV-2 diagnosis.		exclusive to us
36	Johannesen Front Oncol. 2021	Johannesen 2021	<1 year since cancer diagnosis.		included in both

		Sng 2020	Cancer diagnosis/ treatment within the last 12 months or radiological/ biochemical evidence of active/ recurrent cancer.		exclusive to us
		Alpert 2021	Newly diagnosed cancer or on active treatment.		exclusive to us
		Bertuzzi 2021	Presence of localised or metastatic disease at SARS-CoV-2 onset.		exclusive to us
19	Brar 2020 J Clin Oncol.	Brar 2020	Cancer treatment or active surveillance within 6 months of SARS-CoV-2 diagnosis and ongoing management.		included in both
		Klein 2021	Current haematological malignancy, metastatic disease, or cancer-directed systemic medical therapy within the last 6 months.		exclusive to us
21	Dai 2020 Cancer Discov.	Dai 2020	Active treatment within last 3 months.		included in both
44	Lunski Cancer. 2021		Table 5 shows patients with a diagnosis of cancer >1 year consisted of a large proportion (more than 88%) of the population and for the cancer <12 months cohort only univariate data given(deceased or not). This was a multicenter study in the state of Louisiana throughout the Ochsner Health System in both tertiary and nontertiary centers. 2-month retrospective, observational analysis of patients.		included in both
12	Miyashita Ann Oncol. 2020		No explicit definition of active cancer cohort. Data extracted from electronic medical records of Mount Sinai Health System(MSHS) in New York City as aggregate data.	No explicit active cancer cohort.	exclusive to them
14	Tian Lancet Oncol. 2020		No explicit definition of active cancer cohort. Data obtained from data collection forms from the electronic medical records of each designated hospital in Wuhan- Tongji Hospital, Wuhan Union Hospital, Wuhan First Hospital, the Central Hospital of Wuhan, Wuhan Fourth Hospital and Puai Hospital, Fifth Hospital of Wuhan, Wuhan Pulmonary Hospital, Wuhan Jinyintan Hospital, and Wuhan Hankou Hospital.	No multivariable statistics of cancer vs non-cancer	exclusive to them
16	Mehta Cancer Discovery 2020		No explicit definition of active cancer cohort. Data was taken from the Montefiore Health System (New York, NY) and their outcomes measured.	Only comparison within cancer cohort for all clinical outcomes apart for mortality (control group was used for this in Table 4). No multivariate analysis for patient outcomes.	exclusive to them
18	Martínez-López Blood Cancer J. 2020		Table 2 shows the cancer cohort comprises of patients with >18 months since time of diagnosis for MM. Data investigated 167 patients reported from 73 hospitals within the Spanish Myeloma Collaborative Group network in March and April, 2020.	Cancer diagnosis given as >18 months for 112 patients out of 167(significant majority)	exclusive to them
23	Shah 2020 Br J Haematol.		No explicit definition of active cancer cohort . Data taken from the first 80 patients with hematological malignancy to other patients in same time frame from electronic health record at Kings College Hospital, London.	No explicit definition of cancer cohort within past 36 months. Additional issues - supplementary table shows MGUS patients included as part of cohort which is not a malignancy.	exclusive to them
26	Liang 2020 Lancet Oncol.		Study specifies a history of cancer in 18 patients when these patients are looked at on appendix table S1 - several of them have a longer history of cancer >3 year(e.g. 4 and 6 years in several cases)	Cancer diagnosis >36 months and no evidence of active treatment in some.	exclusive to them
29	Yigenoglu J Med Virol. 2021		No explicit definition of active cancer cohort. Data from laboratory-confirmed 188 897 COVID-19 patients diagnosed between 11 March 2020 and 22 June 2020 included in the Republic of Turkey, Ministry of Health database were analyzed retrospectively	No explicit definition of cancer cohort as being from past 36 months.	exclusive to them
37	Rüthrich Ann Hematol. 2021		Table 2 shows that only 193 patients had active disease at time of detection and 59 in remission so only 252 patients fit our criteria out of 435. Data taken from analysis of cancer patients from the LEOSS (Lean European Open Survey on SARS-CoV-2 Infected Patients) registry.	No explicit definition of cancer, patients do not meet the active/remissive cancer criteria at time of study	exclusive to them

38	Montopoli Ann Oncol. 2020		No explicit definition of active cancer cohort. Details of patients with a diagnosis of SARS-CoV-2 infection in the Italian region of Veneto, with or without cancer, were obtained from the following data sources: (i) the Veneto Archive of COVID-19-positive subjects, updated on 1 April 2020, (ii) the Tumor Registry Archive, and (iii) the Regional Medicines Technical Commission.	No explicit timeline given for cancer diagnosis	exclusive to them
39	Meng J Hematol Oncol. 2020		Table 1 shows how multiple patients in the cohort had a cancer diagnosis that was <1 year (21.4%), 1-4 year (20.7%), 5-9 years (38.6%), >10 years (11.1%). Retrospective data taken from 3232 consecutive patients with COVID-19 who were hospitalized between January 18 and March 27, 2020, at Tongji Hospital in Wuhan, China.	Subset of Cancer diagnosis <36 months only 20% of patients and no specific analysis for this subset.	exclusive to them
40	Cattaneo Cancer. 2020		No explicit mention of a cancer cohort with cancer diagnosis <36 months. Table 1 states the breakdown of cancer status with only some of the patients having recent diagnosis or active remission. Data taken as retrospective cohort study of patients managed at the hematology departments of ASST Spedali Civili in Brescia and Grande Ospedale Metropolitano Niguarda in Milan (both in Lombardy)	No explicit definition of cancer diagnosis as being <36 months.	exclusive to them
41	Sun J Natl Cancer Inst Cancer Spectr. 2021		States cohort included patients with active cancer but does not provide a breakdown, no explicit mention of cancer within <3 year. Data taken from EHR of the University of Pennsylvania and cancer diagnoses also confirmed via manual review.	No breakdown of active and remissive cancer seen on study so not able to extract individual outcomes.	exclusive to them
42	Stroppa Future Oncol. 2020		No explicit mention of a cancer cohort with cancer diagnosis <36 months. Univariate analysis only and no mention of it being adjusted. Data from 25 cancer patients infected by COVID-19 admitted at the Piacenza's general hospital from 21 February to 18 March 2020. Outcome from the infection were compared with infected noncancer patients.	No explicit mention of cancer <36 months.	exclusive to them
43	He Leukemia. 2020		No explicit mention of cancer cohort with disease activity in past 36 months. Study included patients with complete remission of cancer. Data taken from accessing standardised forms from electronic medical records of two centres in Wuhan, China	No explicit mention of cancer <36 months.	exclusive to them
45	Joharatnam-Hogan Ther Adv Med Oncol. 2020		Study states history of cancer but no explicit mention of cancer activity in <36 months. Data taken from collected from 30 cancer patients from five London Hospitals and compared to non cancer cohort.	No explicit mention of cancer <36 months.	exclusive to them

Table S5: Comparison of studies included in this review versus studies included in Felice et al. 2022

Felice et al. Reference number	Felice et al. reference name (n=35)	Papers in our study (n=30)	Definition of cancer	Reason for exclusion	Inclusion Status
Reference number not available due to referencing style		Alsakarneh 2024	ICD-10 clinical modification codes of colorectal cancer (C18-C20).		exclusive to us
		Li 2024	Active cancer or those diagnosed with cancer within 1 year of developing community-acquired COVID-19 pneumonia.		exclusive to us
		Starkey 2023	Patients in a national cancer registry.		exclusive to us
		Konermann 2023	Active cancer based on relavant ICD codes.		exclusive to us
		Park 2024	First ICD-10 cancer diagnosis code (C00–99) <1 year before SARS-CoV-2 diagnosis.		exclusive to us
		Turtle 2023	Active cancer diagnosis and treatment.		exclusive to us
		Salvatore 2023	Most recent cancer diagnosis within the last 3 years.		exclusive to us
		Leuva 2022	Active diagnosis of new or previously established cancer in the last 2 years.		exclusive to us
		Hosseini-Moghaddam 2023	Cancer treatment in the last 6 months or diagnosis within the last 1-year.		exclusive to us
		Sullivan 2023	At least two ICD codes for cancer within 1 year before, and anticancer treatment within 3 months before, SARS-CoV-2 diagnosis.		exclusive to us
		Nolan 2023	Any ICD-10 cancer diagnosis recorded during the index SARS-CoV-2 hospitalisation period.		exclusive to us
		Kodde 2023	Presence of any of the three related Elixhauser comorbidities (lymphoma, metastatic cancer, solid tumor).		exclusive to us
		Udovica 2022	Cancer diagnosis/ treatment within the last 6 months or metastatic/ recurrent malignant disease.		exclusive to us
		Bazgir 2022	Cancer treatment in the last 2 months.		exclusive to us
		Plais 2022	Treatment/active surveillance within the last 6 months, or haematological cancer not in complete remission.		exclusive to us
		Chavez-MacGregor 2022	At least two ICD codes for cancer within 1 year before SARS-CoV-2 diagnosis.		exclusive to us
		Abuhelwa 2022	ICD-10 clinical modification codes of active cancer (C00-C99).		exclusive to us
		Serraino 2021	<13 months since cancer diagnosis.		exclusive to us
		Kim 2022	Cancer diagnosed within 1-year before SARS-CoV-2 diagnosis.		exclusive to us
		Zhou 2023	Patients with pre-existing active malignancy who were not in complete remission.		exclusive to us

		Raad 2023	Cancer diagnosis/ treatment within 1-year before SARS-CoV-2 diagnosis.		exclusive to us
		Rugge 2022	Cancer diagnosed within 1-year before SARS-CoV-2 diagnosis.		exclusive to us
		Anantharaman 2021	Received cancer treatment in the 180 days prior to SARS-CoV-2 diagnosis.		exclusive to us
		Johannesen 2021	<1 year since cancer diagnosis.		exclusive to us
		Sng 2020	Cancer diagnosis/ treatment within the last 12 months or radiological/ biochemical evidence of active/ recurrent cancer.		exclusive to us
		Alpert 2021	Newly diagnosed cancer or on active treatment.		exclusive to us
		Bertuzzi 2021	Presence of localised or metastatic disease at SARS-CoV-2 onset.		exclusive to us
	Brar et al., Journal of Clinical Oncology, 2020	Brar 2020	Cancer treatment or active surveillance within 6 months of SARS-CoV-2 diagnosis and ongoing management.		included in both
		Klein 2021	Current haematological malignancy, metastatic disease, or cancer-directed systemic medical therapy within the last 6 months.		exclusive to us
	Dai et al., Cancer Discovery, 2020	Dai 2020	Active treatment within last 3 months.		included in both
	Haase et al., Acta Anaesthesiologica Scandinavica, 2021		Not provided.	Cancer diagnosis time unknown	exclusive to them
	Meng et al., Journal of Hematology & Oncology, 2020		All patients were identified according to the latest diagnosis and treatment protocol for COVID-19 (trial version 7) issued by the General Office of the National Health Commission. No explicit definition of cancer.	No active malignancy.	exclusive to them
	Sun et al., medRxiv., 2020		Cancer patients met at least 1 of 3 criteria: 1) 3 or more International Classification of Diseases 10th Revision (ICD-10) billing codes for an invasive cancer, 2) inclusion in the Penn Medicine Cancer Registry, and 3) 1 visit within a Cancer Service Line clinic. Cancer diagnoses were confirmed by manual chart review.	Cancer diagnosis time unknown for cases in criteria 2 and 3.	exclusive to them
	Nogueira et al., Journal of Clinical Medicine, 2020		Not provided.	N/A	exclusive to them
	Zandkarimi et al., International Journal of Health Policy and Management, 2020		Not provided.	Wrong comparator	exclusive to them
	Zhao et al., Infectious Diseases of Poverty, 2020		N/A	Wrong comparator	exclusive to them
	Harrison et al., PLOS Medicine, 2020		Any malignancy, including lymphoma and leukemia, except malignant neoplasm of skin. Comorbidities are based on ICD-10 codes from 2015.	No active malignancy	exclusive to them
	Gupta et al., JAMA Internal Medicine, 2020		Active cancer.	Wrong publication type (Erratum)	exclusive to them
	Ganatra et al., Journal of the National Comprehensive Cancer Network, 2020		Patients were determined to have a history of cancer if they had any active or prior solid or hematologic malignancy, regardless of the need for therapy. Non-invasive cancers, including non-melanomatous skin cancer, in-situ carcinoma, or precursor	No active malignancy	exclusive to them

			hematologic neoplasms, were not included in the definition of cancer.		
	Westblade et al., Cancer Cell, 2020		We defined patients with active cancer as having received cancer-directed therapy (e.g., chemotherapy, targeted therapy, immunotherapy, radiotherapy, or surgery) within six months of admission for COVID-19 or who were receiving active surveillance for their malignancy (e.g., patient with chronic lymphocytic leukemia or prostate cancer).	Defines active cancer as active surveillance within 6 months or treatment within 6 months, while no active cancer, the non cancer control, is defined as everyone else, hence, someone who had chemotherapy for cancer 181 days before admission is treated the same as a healthy individual who has never had cancer.	exclusive to them
	Wang et al., Journal of Hematology & Oncology, 2020		The study was designed from a register of patients with SMM and MM in any phase of the response, currently receiving treatment or follow-up at the Mount Sinai Hospital.	No appropriate control group	exclusive to them
	Cherri et al., Therapeutic Advances in Medical Oncology, 2020		oncological patients (i.e. patients with active or previous oncological disease)	COVID-19 not laboratory confirmed using RT-PCR	exclusive to them
	Görgülü et al., SN Comprehensive Clinical Medicine, 2020		Not provided.	COVID-19 not laboratory confirmed using RT-PCR or RAT	exclusive to them
	Jiménez et al., BMJ Open, 2020		Not provided.	previous malignancy/no active malignancy	exclusive to them
	Thompson et al., Epidemiology and Infection, 2020		Not provided.	Ambiguous cancer cohort	exclusive to them
	Li et al., Leukemia, 2020		Solid cancers and hematological cancers were included. Cancer remission was defined as complete remission for $\geq$1 year	cancer diagnosis time unknown	exclusive to them
	Shoumariyeh et al., Cancer Medicine, 2020		hospitalized patients at the UHF with an active hematological, solid cancer or cancer in remission	Remission patients included	exclusive to them
	Mehta et al., Cancer Discovery, 2020		These records were cross-referenced with an existing cancer database, and a retrospective review was done by senior authors to extract additional data on patients with a history of malignancy. Cases identified as having benign neoplasms were excluded.	cancer diagnosis time unknown	exclusive to them
	Rogado et al., Clinical & Translational Oncology, 2020		Not provided.	No appropriate control group	exclusive to them
	Zhang et al., Frontiers in Pharmacology, 2020b		We retrospectively review 1069 medical records admitted to Medical Oncology department at Hospital Universitario Infanta Leonor of Madrid, from February 1, 2020, to April 7, 2020.	Cancer diagnosis time unknown	exclusive to them
	Sorouri et al., Infectious Agents and Cancer, 2020		Primary cancer characteristics and detailed treatment information were also obtained by review of the patients' past medical records.	COVID-19 not laboratory confirmed using RT-PCR	exclusive to them
	Lunski et al., Cancer, 2021		International Classification of Diseases, Tenth Revision codes were used to identify patients with active cancer or a history of cancer.	Data extraction completed.	exclusive to them
	Atalla et al., International Journal of Clinical Practice, 2021		Not provided.	Wrong outcomes	exclusive to them
	Cheng et al., Acta Oncologica (Stockholm, Sweden), 2020		Not provided.	Data extraction completed.	exclusive to them
	Song et al., Allergy, 2021		Not provided.	N/A	exclusive to them

	Liang et al., JAMA Internal Medicine, 2020		Not provided.	Wrong study design	exclusive to them
	Bauer et al., Journal of Clinical Hypertension (Greenwich, Conn.), 2021		Not provided.	COVID-19 not laboratory confirmed using RT-PCR or RAT	exclusive to them
	Tian et al., The Lancet. Oncology, 2020		We included all adult patients (aged ≥18 years) admitted between Jan 13 and March 18, 2020, with laboratory-confirmed SARS-CoV-2 infection by RT-PCR who had any type of malignant solid tumour and haematological malignancies.	Cancer diagnosis time unknown	exclusive to them
	Yigenoglu et al., Journal of Medical Virology, 2021		All COVID-19 patients with hematological malignancy (n =740) were included in the study and age, sex, and comorbidity matched COVID-19 patients without cancer (n=740) at 1:1 ratio was used for comparison.	Cancer diagnosis time unknown	exclusive to them
	Shah et al., British Journal of Haematology, 2020		Our aim was to compare the first 80 patients with a haematological malignancy with all other patients admitted to our hospital with COVID-19 in the same time frame, to precisely define their relative risk and identify factors that increase mortality within this subgroup.	Wrong publication type (Correspondence)	exclusive to them
	Sanchez-Pina et al., European Journal of Haematology, 2020		Haematological malignancy patients who were treated at a haematological unit.	Cancer diagnosis time unknown	exclusive to them
	Passamonti et al., The Lancet. Haematology, 2020		Inclusion criteria were the presence of a WHO-defined haematological malignancy and symp tomatic and laboratory-confirmed SARS-CoV-2 infection, tested by RT-PCR on nasopharyngeal swabs.	Data extraction completed.	exclusive to them
	Cattaneo et al., Cancer, 2020		The roles of age, sex, and hematologic disease in the risk of COVID-19 infection were analysed through a comparison of SARS-CoV-2–infected patients diagnosed at the hematology department in Brescia with all 4244 patients seen during 2019 at the same department.	Wrong patient population	exclusive to them

Table S6: Comparison of studies included in this review versus studies included in Han et al. 2022

Han et al. Reference number	Han et al. Reference names (n=57)	Papers in our study (n=30)	Definition of cancer	Reason of exclusion	Inclusion Status
		Alsakarneh 2024	ICD-10 clinical modification codes of colorectal cancer (C18-C20).		exclusive to us
		Li 2024	Active cancer or those diagnosed with cancer within 1 year of developing community-acquired COVID-19 pneumonia.		exclusive to us
		Starkey 2023	Patients in a national cancer registry.		exclusive to us
		Konermann 2023	Active cancer based on relavant ICD codes.		exclusive to us
		Park 2024	First ICD-10 cancer diagnosis code (C00–99) <1 year before SARS-CoV-2 diagnosis.		exclusive to us
		Turtle 2023	Active cancer diagnosis and treatment.		exclusive to us
		Salvatore 2023	Most recent cancer diagnosis within the last 3 years.		exclusive to us
		Leuva 2022	Active diagnosis of new or previously established cancer in the last 2 years.		exclusive to us
		Hosseini-Moghaddam 2023	Cancer treatment in the last 6 months or diagnosis within the last 1-year.		exclusive to us
		Sullivan 2023	At least two ICD codes for cancer within 1 year before, and anticancer treatment within 3 months before, SARS-CoV-2 diagnosis.		exclusive to us
		Nolan 2023	Any ICD-10 cancer diagnosis recorded during the index SARS-CoV-2 hospitalisation period.		exclusive to us
		Kodde 2023	Presence of any of the three related Elixhauser comorbidities (lymphoma, metastatic cancer, solid tumor).		exclusive to us
		Udovica 2022	Cancer diagnosis/ treatment within the last 6 months or metastatic/ recurrent malignant disease.		exclusive to us
		Bazgir 2022	Cancer treatment in the last 2 months.		exclusive to us
		Plais 2022	Treatment/active surveillance within the last 6 months, or haematological cancer not in complete remission.		exclusive to us
		Chavez-MacGregor 2022	At least two ICD codes for cancer within 1 year before SARS-CoV-2 diagnosis.		exclusive to us
		Abuhelwa 2022	ICD-10 clinical modification codes of active cancer (C00-C99).		exclusive to us
		Serraino 2021	<13 months since cancer diagnosis.		exclusive to us
		Kim 2022	Cancer diagnosed within 1-year before SARS-CoV-2 diagnosis.		exclusive to us
		Zhou 2023	Patients with pre-existing active malignancy who were not in complete remission.		exclusive to us
		Raad 2023	Cancer diagnosis/ treatment within 1-year before SARS-CoV-2 diagnosis.		exclusive to us
		Rugge 2022	Cancer diagnosed within 1-year before SARS-CoV-2 diagnosis.		exclusive to us
		Anantharaman 2021	Received cancer treatment in the 180 days prior to SARS-CoV-2 diagnosis.		exclusive to us
		Johannesen 2021	<1 year since cancer diagnosis.		exclusive to us
		Sng 2020	Cancer diagnosis/ treatment within the last 12 months or radiological/ biochemical evidence of active/ recurrent cancer.		exclusive to us

		Alpert 2021	Newly diagnosed cancer or on active treatment.		exclusive to us
		Bertuzzi 2021	Presence of localised or metastatic disease at SARS-CoV-2 onset.		exclusive to us
		Brar 2020	Cancer treatment or active surveillance within 6 months of SARS-CoV-2 diagnosis and ongoing management.		exclusive to us
		Klein 2021	Current haematological malignancy, metastatic disease, or cancer-directed systemic medical therapy within the last 6 months.		exclusive to us
		Dai 2020	Active treatment within last 3 months.		exclusive to us
31	Argenziano et al, BMJ, 2020		Active cancer' determined via medical chart review.	No multivariate analysis of active cancer vs. non-cancer.	exclusive to us
33	Benelli et al, PLoS One, 2020		previous and active malignancy	cancer as comorbidity (no reasonable adjustment for confounding).	exclusive to them
42	Feng et al, Am J Respir Crit Care Med, 2020		given as "malignancy" in table 2	cancer as comorbidity (no reasonable adjustment for confounding)	exclusive to them
43	Guan et al, N Engl J Med, 2020		"Any type of cancer”, listed as a comorbidity in table 2	cancer as comorbidity (no reasonable adjustment for confounding)	exclusive to them
50	Li et al, J Allergy Clin Immunol, 2020		No definition given, listed as a comorbidity under "tumour" in table 1	cancer as comorbidity (no reasonable adjustment for confounding)	exclusive to them
51	Liang et al, Clin Infect Dis, 2020		Listed as a comorbidity in table 1 as "cancer" no further specifications	<10 patients with COVID and cancer	exclusive to them
54	Myers et al, Jama, 2020		No definition given, listed as a comorbidity under "malignancy" in table	No multivariate analysis of active cancer vs. non-cancer.	exclusive to them
58	Petrilli et al, BMJ, 2020		Cancer' determined via medical chart review.	Cancer diagnosis time unknown.	exclusive to them
66	Wang et al, Jama, 2020		no definition given	Wrong outcomes	exclusive to them
74	Zhang et al, Clin Microbiol Infect, 2020		No definition given, listed as a comorbidity under "malignant tumour" in table 1	Cancer as comorbidity	exclusive to them

Table S7: Outcome definitions within 30 included studies

Reference	Hospitalisation		Severity			ICU admission		Mortality		Variables adjusted for in multivariate analysis			
	Reported?	Definition	Reported?	Assessment	Definition	Reported?	Definition	Reported?	Definition (duration; cause)	Age	Sex	Comorbidities	Other
Alsakarneh 2024	*	All-cause hospitalisation within 30-days of breakthrough SARS-CoV-2 infection.		NA	NA	*	NR	*	30-day mortality; all-cause.			Comorbidities related to SARS-CoV-2 morbidity and mortality.	Race, ethnicity, social determinants of adverse health outcomes, behavioural factors (tobacco smoking, alcohol abuse), COVID vaccine type, cancer treatment types.
Li 2024		NA	Multivariate analysis not reported.	Clinical characteristics.	Any of: (1) shortness of breath with a respiratory rate >30 breaths per minute; (2) pulse oxygen saturation ≤93% at rest; (3) PaO2/FiO2 ≤300 mmHg.	All study patients were admitted to ICU.	NR	*	NR; death of patients within the ICU, including those expected to die following the discontinuation of treatment.				Acute Physiology and Chronic Health Evaluation (APACHE) II score.
Starkey 2023	*	SARS-CoV-2 inpatient hospitalisation episode lasting more than one day.		NA	NA	*	Intensive care admission following a SARS-CoV-2 hospitalisation.	*	28 days; all-cause.				Deprivation, ethnicity.
Konermann 2023		NA		NA	NA		NA	Multivariate analysis not reported for active cancer vs. no cancer.	Discharge status; death associated with viral pneumonia (J12), pneumonia (J18), respiratory insufficiency			Diabetes mellitus, dyslipidemia, obesity, heart failure, coronary artery disease, liver cirrhosis/fibrosis, cerebrovascular disease, chronic pulmonary disease, and chronic kidney disease.	

	[redacted]		[redacted]			[redacted]			(J96), or sepsis (A41).	[redacted]			[redacted]
Park 2024	*	NR	*	Need for respiratory and/or organ support.	Hospitalised and requiring non-invasive ventilation, high-flow oxygen, intubation and mechanical ventilation, or ventilation combined with additional organ support such as dialysis and extracorporeal membrane oxygenation.	[redacted]	NA	*	NR; NR.			Charlson Comorbidity Index.	SARS-CoV-2 vaccination status, household income.
Turtle 2023	[redacted]	NA	*	Need for respiratory support.	Requiring non-invasive ventilation.	*	ICU admission; Requiring invasive ventilation.	*	28-day mortality; all-cause in-hospital mortality.			NR	Deprivation, ethnicity, socioeconomic deprivation, vaccination status.
Salvatore 2023	*	Hospitalisation between 14 days prior to and 30 days after SARS-CoV-2 diagnosis.	Severity reported as a composite outcome including hospitalisation, which was already reported as a separate outcome and included in meta-analysis for 'hospitalisation'.	Survival and hospitalisation status.	Composite outcome: hospitalisation and/or ICU admission and/or all-cause mortality during SARS-CoV-2 infection.	*	ICU admission between 14 days prior to and 30 days after SARS-CoV-2 diagnosis.	*	60-day mortality; all-cause.			Comorbidity Index Score.	Neighborhood disadvantage index, race/ethnicity.
Leuva 2022	[redacted]	NA	[redacted]	NA	NA	[redacted]	NA	*	60-day mortality; all-cause.			Elixhauser comorbidity index.	Race, body mass index, smoking status, rurality of normal residence, time during the pandemic.
Hosseini-Moghaddam 2023	Insufficient studies reporting adjusted hazard ratios	14-day all-cause hospitalisation.	[redacted]	NR	NR	Adjusted hazard ratios reported separately for solid and	21-day ICU admission.	Adjusted hazard ratios reported separately for solid and	28-day mortality; all-cause.			NR	SARS-CoV-2 vaccination status, past health care utilisation,

	for hospitalisation to perform meta-analysis for this outcome.					haematological cancers. Insufficient studies reporting adjusted hazard ratios by cancer subtype to perform meta-analysis for this outcome.		haematological cancers. Insufficient studies reporting adjusted hazard ratios by cancer subtype to perform meta-analysis for this outcome.					income, rurality, Public Health Unit region, Ontario Marginalisation Index, 2016 Canada census variables.
Sullivan 2023	*	Hospitalisation within 30 days of SARS-CoV-2 diagnosis.	*	Need for respiratory support.	Requiring mechanical ventilation within 30 days of SARS-CoV-2 diagnosis.	*	ICU admission within 30-days of SARS-CoV-2 diagnosis.	*	30-day mortality; NR.		**	Charles-Deyo comorbidity index score	SARS-CoV-2 diagnosis period, race and ethnicity, severe obesity (BMI≥40), recent skilled scaled nursing facility stay, insurance type, region.
Nolan 2023		NA		NA	NA	*	NR	*	in-hospital; all-cause mortality.			Weighted Elixhauser comorbidity index.	Race, ethnicity, insurance status, BMI, smoking status, vaccination status, year of the pandemic.
Kodde 2023		NA	*	Need for respiratory support.	Requiring mechanical ventilation.		NA	*	NR; in-hospital mortality.			Weighted Elixhauser comorbidity index.	
Udovica 2022		NA	Multivariate analysis not reported.	Need for respiratory support.	SARS-CoV-2 associated with hypoxia necessitating conventional oxygen supplementation to keep blood oxygen saturation ≥94%.	Multivariate analysis not reported.	Requiring treatment in an ICU for respiratory or multiorgan failure.	*	NR; SARS-CoV-2-specific (death while requiring > 6L/min oxygen; or death with signs of pulmonary manifestation of COVID-19 on CXR, or death in the ICU due to respiratory or multiorgan failure)			Chronic cardiovascular disease, chronic respiratory disease, moderate or severe liver disease, severe chronic kidney disease, diabetes, dementia, neurological diseases, obesity.	ECOG performance status.

Bazgir 2022		NA		NA	NA	*	NR	*	NR; in-hospital mortality.			NR	
Plais 2022		NA		NA	NA		NA	*	60-day mortality; all-cause.			BMI, diabetes mellitus, chronic respiratory disease, congestive heart failure, organ transplant, immunosuppressive therapy, HIV.	Antimicrobial treatment, hospitalisation within the last 3 months, SAPS II and SOFA scores, and PaO2 at baseline.
Chavez-MacGregor 2022	*	Hospitalisation within 30-days of SARS-CoV-2 diagnosis.	*	Need for respiratory support.	Requiring mechanical ventilation within 30-days of SARS-CoV-2 diagnosis.	*	ICU admission within 30-days of SARS-CoV-2 diagnosis.	*	30 day mortality; all-cause.			NR	Race, ethnicity, severe obesity, skilled nursing facility stay within 3 months before SARS-CoV-2 diagnosis, 2020 calendar quarters, insurance type, and region.
Abuhelwa 2022		NA	*	Need for respiratory support.	Requiring mechanical ventilation.		NA	*	NR; all-cause in-hospital.				Race, admission type, admission day, hospital bed size, hospital teaching status, hospital region, insurance status, median annual income.
Serraino 2021		NA		NA	NA	*	NR	*	NR; all-cause.				
Kim 2022	*	Hospitalisation within 30-days of SARS-CoV-2 diagnosis.	*	Need for respiratory support.	Requiring mechanical ventilation within 30-days of SARS-CoV-2 diagnosis.	*	ICU admission within 30-days of SARS-CoV-2 diagnosis.	*	30-day mortality; all-cause.			Chronic pulmonary disease, cardiovascular, cerebrovascular, peripheral vascular, diabetes, obesity, liver, renal diseases..	Race/ethnicity, insurance type, regions.
Zhou 2023		NA		NA	NA	*	Composite outcome: intubation and/or ICU admission and/or 30-day mortality.	Only reported as a composite outcome with intubation and/or ICU admission.	30-day mortality; NR.			Charlson's comorbidity index, number of comorbidities, past comorbidities.	Medication treatments

Raad 2023	Multivariate analysis not reported.	NR	Multivariate analysis not reported for cancer vs. non-cancer.	Need for respiratory support.	Requiring non-invasive ventilation at diagnosis.	Multivariate analysis not reported.	NR	*	30-day mortality; all-cause.	[redacted]	[redacted]	[redacted]	Country, SARS-CoV-2 treatment.
Rugge 2022	*	NR	[redacted]	NA	NA	*	NR	*	NR; all-cause.			Respiratory, others, none.	[redacted]
Anantharaman 2021	*	45-day inpatient hospitalisation.	*	Need for respiratory support.	Requiring non-invasive mechanical ventilation within 45-days of SARS-CoV-2 diagnosis.	*	45-day ICU admission.	*	As of study end date; all-cause.			Charlson comorbidity index, hypertension, diabetes.	Race/ethnicity, body mass index, smoking status, neighborhood deprivation index, week of SARS-CoV-2 diagnosis.
Johannesen 2021	*	Composite outcome: admission to hospital due to SARS-CoV-2 and/or death.	[redacted]	NA	NA	[redacted]	NA	Only reported as a composite outcome with admission to hospital due to SARS-CoV-2.	As of study end date; SARS-CoV-2-specific (cause of death registry).			[redacted]	[redacted]
Sng 2020	[redacted]	NA	[redacted]	NA	NA	[redacted]	NA	*	3-month mortality; all-cause.		[redacted]	Cardiovascular disease, chronic kidney disease, hypertension, cerebrovascular disease.	Ethnicity.
Alpert 2021	[redacted]	NA	*	Clinical and biochemical characteristics.	Sepsis defined as more than one of the following: temperature >100.4 degrees Farenheit; heart rate >90 bpm; respiratory rate >20 bpm; white blood cell count <4,000 or >12,000 cells/lL.	*	NR	*	NR with median follow up time of 18 months (IQR 7.8-44); all-cause.			Number of comorbidities.	[redacted]
Bertuzzi 2021	[redacted]	NA	[redacted]	NA	NA	Multivariate analysis not reported.	NR	*	NR with median follow up time of 12 months (IQR 0-76); all-cause.		[redacted]	Coronary artery disease.	Lactate dehydrogenase, procalcitonin, PaO2/FiO2, cigarette smoking.
Brar 2020	[redacted]	NA	[redacted]	NA	NA	*	Composite outcome: ICU admission and/or	*	As of study end date; all-cause.			Diabetes, Hypertension, COPD, coronary artery disease, heart failure, obesity.	Ethnicity, smoking history.

	■		■				intubation and/or death.						
Klein 2021	■	NA	*	Clinical characteristics.	Acute respiratory distress syndrome.	*	NR	*	As of study end date; all-cause mortality or discharge to hospice.	■	■	Immunosuppressive status, cardiovascular comorbidities, pulmonary comorbidities, diabetes.	■
Dai 2020	■	NA	Insufficient studies reporting adjusted hazard ratios for severe disease to perform meta-analysis for this outcome.	Based on the 5th edition of the '2019 Novel Coronavirus Disease Diagnostic Criteria' published by the National Health Commission in China.	Adults who meet any one of the following: Shortness of breath or Respiratory Rate >30 breaths/minute; Oxygen saturation <93% at rest; Arterial oxygen partial pressure (PaO2)/ fraction of inspired oxygen (FiO2)<300mmHg; Lung imaging shows a substantial progression of lesions (greater than 50%) within 24-48 hours.	*	NR	*	As of study end date; all-cause mortality.			Diabetes, hypertension, COPD.	Smoking status.

*, Included in meta-analysis; **, All patients included in Sullivan et al. 2023 were female; NA, Not applicable; NR, Not reported; Black box, No; White box, Yes.

Table S8: Comparison of demographic characteristics in cancer vs non-cancer patients in the 30 included studies

Reference	Patients with SARS-CoV-2 and cancer						Patients with SARS-CoV-2 without cancer						Total (*n*)
	n	Age				Male (%)	*n*	Age				Male (%)	
		Measure of centre	Centre value	Measure of spread	Spread value			Measure of centre	Centre value	Measure of spread	Spread value		
Alsakarneh 2024	15416	Mean	69.6	SD	12.5	51.4	15416	Mean	70.2	SD	12.2	50.1	30832
Li 2024	77	Median	69	IQR	59-77	74	1788	Median	76	IQR	68-84	72.5	1865
Starkey 2023	127322	NR	NR	NR	NR	50.48	15801004	NR	NR	NR	NR	43.05	15928326
Konermann 2023	1513	Median	Wild-Type: 75; Omicron: 74	IQR	Wild-Type: 65-82; Omicron: 64 - 81	Wild-Type: 58; Omicron: 62	24912	Median	Wild-Type: 70; Omicron: 72	IQR	Wild-Type: 54-82; Omicron: 53-83	Wild-Type: 52; Omicron: 49	26425
Park 2024	40334	NR	NR	NR	NR	NR	397050	NR	NR	NR	NR	44.6	437384
Turtle 2023	5116	Median	72	IQR	62.0-79.7	58.1	134598	Median	69.5	IQR	53.4-82.0	55.3	139714
Salvatore 2023	6143	NR	NR	NR	NR	NR	38267	NR	NR	NR	NR	NR	44410
Leuva 2022	10355	NR	NR	NR	NR	NR	10696	NR	NR	NR	NR	NR	21051
Hosseini-Moghaddam 2023	8378	Mean	59.61	SD	18.05	40.6	456196	Mean	45.95	SD	18.24	48.96	464574
Sullivan 2023	2200	Median	65	IQR	55-74	0	22000	Median	65	IQR	55-74	0	24200
Nolan 2023	7141	NR	NR	NR	NR	55	90700	NR	NR	NR	NR	49.2	97841
Kodde 2023	1625	Mean	72.2	SD	11.8	58.2	27659	Mean	68	SD	17.6	52.1	29284
Udovica 2022	89	Median	74	Range	51-95	50.6	156	Median	76	Range	26-97	54.6	245
Bazgir 2022	64	Mean	63.9	SD	2.5	53.1	256	Mean	62.1	SD	0.8	52.7	320
Plais 2022	105	Mean	69	NR	65-73	68.6	315	Mean	67	NR	59-75	69.4	420
Chavez-MacGregor 2022	14287	Median	66	IQR	57-75	47.9	493020	Median	48	IQR	32-61	44.5	507307
Abuhelwa 2022	27760	Mean	70.92	95% CI	70.52-71.32	56.57	1022285	Mean	64.57	95% CI	64.41-64.74	52.7	1050045
Serraino 2021	466	NR	NR	NR	NR	46.9	38268	NR	NR	NR	NR	46.7	38734
Kim 2022	10426	Median	66	IQR	57-76	47.3	253179	Median	46	IQR	32-60	42.7	263,605
Zhou 2023	142	Median	63.82	IQR	51.01-78.62	45.07	5947	Median	45.22	IQR	27.62-62.33	49.87	6089
Raad 2023	1115	Median	61	Range	18-100	45	2851	Median	50	Range	18-100	47	3966
Rugge 2022	324	Median	73	IQR	62-82.25	53.4	20777	Median	51	IQR	36-66	40.1	21101
Anantharaman 2021	33	NR	NR	NR	NR	45	4380	NR	NR	NR	NR	48	4413
Johannesen 2021	53	NR	NR	NR	NR	NR	7841	NR	NR	NR	NR	NR	7894
Sng 2020	94	Median	71	IQR	62-80	66	226	Median	70.5	IQR	60-80	67	320
Alpert 2021	421	Mean	69.2	SE	0.7	59.4	5135	Mean	63.8	SE	0.2	56.6	5556
Bertuzzi 2021	46	Mean	71	95% CI	67-74	67.4	511	Mean	65	95% CI	64-66	67.3	557
Brar 2020	117	Median	72.5	IQR	64.2-79.9	54.7	468	Median	71.2	IQR	62.1-79.6	54.7	585
Klein 2021	77	NR	NR	NR	NR	NR	324	NR	NR	NR	NR	NR	401

Dai 2020	31	Mean	67.26	SD	8.4	80.6	186	NR	NR	NR	NR	NR	217

NR, Not reported.

Table S9: Comparison of comorbidity status in cancer vs non-cancer patients in the 30 included studies

Reference	Total patients with SARS-CoV-2 and cancer (n)	Total patients with SARS-CoV-2 and without cancer (n)	Total number of patients (n)	Smoking Cancer (%)	Smoking Control (%)	Hypertension Cancer (%)	Hypertension Control (%)	Diabetes Cancer (%)	Diabetes Control (%)	CVD Cancer (%)	CVD Control (%)	Cerebrovascular Disease Cancer (%)	Cerebrovascular Disease Control (%)	Chronic Liver Disease Cancer (%)	Chronic Liver Disease Control (%)	CKD Cancer (%)	CKD Control (%)	Chronic Lung Disease Cancer (%)	Chronic Lung Disease Control (%)	Immunodeficiency Cancer (%)	Immunodeficiency Control (%)
Alsakarneh 2024	15416	15416	30832	4.8	4.8	64.6	68.5	27.6	29.7	HF: 12.7; IHD: 26.8	HF: 13.1; IHD: 26.9	12.2	13	22.4	21.7	18.7	19.3	24.9	25.7	HIV: 1.2	HIV: 1.2
Li 2024	77	1788	1865	24.7	26.5	40.3	55.9	22.1	29.9	NR	NR	NR	NR	2.6	1	6.5	6.3	13	12.4	NR	NR
Starkey 2023	127322	15801004	15928326	NR	NR	NR	NR	NR	NR	NR	NR	NR	NR	NR	NR	NR	NR	NR	NR	NR	NR
Konermann 2023 WT-D	777	14998	15775	NR	NR	NR	NR	21	24	15	13	5.8	6.6	1.4	0.6	18	16	13	9.3	NR	NR
Konermann 2023 Omicron	736	9914	10650	NR	NR	NR	NR	19	19	14	14	4.5	6.8	2	0.9	15	13	9.9	8.5	NR	NR
Park 2024	40334	397050	NR	NR	NR	NR	NR	NR	NR	NR	NR	NR	NR	NR	NR	NR	NR	NR	NR	NR	NR
Turtle 2023	5116	134598	139714	31.7	25.1	40.1	42.4	23.2	27	25.8	26.4	NR	NR	4.5	2.8	15.4	14	18.5	13.7	100	0
Salvatore 2023	6143	38267	44410	NR	NR	NR	NR	NR	NR	NR	NR	NR	NR	NR	NR	NR	NR	NR	NR	NR	NR
Leuva 2022	10355	10696	21051	NR	NR	NR	NR	NR	NR	NR	NR	NR	NR	NR	NR	NR	NR	NR	NR	NR	NR
Hosseini-Moghaddam 2023	8378	456196	464574	NR	NR	50.4	23.9	29.5	15.2	17.9	5.8	2.5	0.9	1.5	0.2	9.3	2.6	24.3	18.4	1.5	0.4
Sullivan 2023	2200	22000	NR	NR	NR	NR	NR	NR	NR	NR	NR	NR	NR	NR	NR	NR	NR	NR	NR	NR	NR
Nolan 2023	7141	90700	97841	50.6	36.9	NR	NR	NR	NR	NR	NR	NR	NR	NR	NR	NR	NR	NR	NR	NR	NR
Kodde 2023	1625	27659	29284	NR	NR	58.6	NR	29.9	NR	24.7	NR	NR	NR	7.1	NR	NR	NR	NR	NR	NR	NR
Udovica 2022	89	156	245	NR	NR	NR	NR	15.7	28.8	52.8	45.5	NR	NR	11.2	6.4	11.2	10.3	24.7	15.4	NR	NR
Bazgir 2022	64	256	320	0	2.7	32.8	37.9	17.2	25	21.9	29.7	3.1	3.1	NR	NR	5.5	7.8	7.8	7.8	NR	NR

Plais 2022	105	315	420	NR	NR	NR	NR	22.9	34	9.5	9.2	NR	NR	1	2.5	11.4	10.2	7.6	3.8	24.8	5.4
Chavez-MacGregor 2022	14287	493020	507307	NR	NR	NR	NR	NR	NR	NR	NR	NR	NR	NR	NR	NR	NR	NR	NR	NR	NR
Abuhelwa 2022	27760	1022285	1050045	36.42	26.73	69.81	67.75	34.53	41.08	22.24	18.27	NR	NR	4.76	4.34	17.26	14.68	NR	NR	NR	NR
Serraino 2021	466	38268	38734	NR	NR	NR	NR	21	8.18	45.4	18.8	NR	NR	NR	NR	NR	NR	13.7	5.19	NR	NR
Kim 2022	10426	253179	263605	NR	NR	NR	NR	35	16.8	49.7	19.6	19	6.2	22.4	7.2	25.7	7.2	42.2	24.7	NR	NR
Zhou 2023	142	5947	6089	NR	NR	38.02	14.2	7.74	2.21	NR	NR	6.33	1.88	2.11	0.48	0.7	0.25	4.22	1.26	NR	NR
Raad 2023	1115	2851	3966	38	17	49	36	27	23	12	12	NR	NR	NR	NR	NR	NR	7	9	NR	NR
Rugge 2022	324	20777	21101	NR	NR	NR	NR	NR	NR	NR	NR	NR	NR	NR	NR	NR	NR	NR	NR	NR	NR
Anantharaman 2021	33	4380	4413	39	25	42	13	18	13	NR	NR	NR	NR	NR	NR	NR	NR	NR	NR	NR	NR
Johannesen 2021	53	7841	7894	NR	NR	NR	NR	NR	NR	NR	NR	NR	NR	NR	NR	NR	NR	NR	NR	NR	NR
Sng 2020	94	226	320	7	6	39	54	26	32	19	26	13	16	3	2	13	12	15	21	4	6
Alpert 2021	421	5135	5556	NR	NR	53	32.7	33	22.2	42	25.3	NR	NR	6.2	2.1	16.6	11	15.2	8.3	NR	NR
Bertuzzi 2021	46	511	557	19.6	8	45.7	51.6	26.1	24.5	NR	NR	NR	NR	NR	NR	NR	NR	NR	NR	NR	NR
Brar 2020	117	468	585	33.4	27.7	53	57.5	32.5	29.1	NR	NR	NR	NR	NR	0.4	2.6	4.9	NR	NR	0	0.6
Klein 2021	77	324	401	NR	NR	NR	NR	NR	40.7	NR	34.3	NR	NR	NR	3.7	NR	25.6	NR	18.2	NR	8.6
Dai 2020	31	186	217	48.39	NR	29.03	NR	12.9	NR	12.9	NR	6.45	NR	NR	NR	NR	NR	9.68	NR	NR	NR
**Total**	281270	18876411		15,852	342,280	27,593	863,570	18,364	579,964	15,411	310,907	2,294	21,623	4,197	67,629	9,400	203,475	7,820	170,059	5,271	1,886
**Total Percentage**				5.60%	1.80%	9.80%	4.60%	6.50%	3.10%	5.50%	1.60%	0.80%	0.10%	1.50%	0.40%	3.30%	1.10%	2.80%	0.90%	1.90%	0.00%

NR, Not reported; HF, Heart failure; IHD, Ischaemic heart disease; WT-D, Wild-Type to Delta period.

Table S10: Breakdown of cancer subtypes across 30 included studies

Reference	Total patients with SARS-CoV-2 and cancer (n)	Patients with cancer subtypes (n)																
		Haematological (total)	Solid (total)	Metastatic	Non-Metastatic	Breast	CNS	Gynaecological	Head and Neck	Hepato-Pancreato-Biliary	Kidney	Lower GI	Sarcoma	Skin Cancer	Thoracic	Upper GI	Urological	Other
Alsakarneh 2024	15416	NR	15416	NR	NR	NR	NR	NR	NR	NR	NR	15416	NR	NR	NR	NR	NR	NR
Li 2024	77	NR	NR	NR	NR	NR	NR	NR	NR	NR	NR	NR	NR	NR	NR	NR	NR	NR
Starkey 2023	127322	NR	NR	NR	NR	NR	NR	NR	NR	NR	NR	NR	NR	NR	NR	NR	NR	NR
Konermann 2023	1513	341	1172	NR	NR	NR	NR	NR	NR	NR	NR	NR	NR	NR	NR	NR	NR	NR
Park 2024	40334	NR	NR	NR	NR	NR	NR	NR	NR	NR	NR	NR	NR	NR	NR	NR	NR	NR
Turtle 2023	5116	NR	NR	NR	NR	NR	NR	NR	NR	NR	NR	NR	NR	NR	NR	NR	NR	NR
Salvatore 2023	6143	NR	NR	NR	NR	NR	NR	NR	NR	NR	NR	NR	NR	NR	NR	NR	NR	NR
Leuva 2022	10355	1842	8513	NR	NR	NR	NR	NR	NR	NR	NR	475	NR	NR	728	NR	3224	NR
Hosseini-Moghaddam 2023	8378	1193	7185	NR	NR	1272	47	1797	698	71	271	676	NR	125	321	132	1336	439
Sullivan 2023	2200	NR	2200	NR	NR	2200	NR	NR	NR	NR	NR	NR	NR	NR	NR	NR	NR	NR
Nolan 2023	7141	2482	4337	NR	NR	611	NR	NR	NR	NR	NR	1062	NR	NR	732	NR	628	NR
Kodde 2023	1625	NR	NR	NR	NR	NR	NR	NR	NR	NR	NR	NR	NR	NR	NR	NR	NR	NR
Udovica 2022	89	NR	NR	NR	NR	NR	NR	NR	NR	NR	NR	NR	NR	NR	NR	NR	NR	NR
Bazgir 2022	64	NR	NR	NR	NR	NR	NR	NR	NR	NR	NR	NR	NR	NR	NR	NR	NR	NR
Plais 2022	105	51	54	NR	NR	NR	NR	NR	NR	NR	NR	NR	NR	NR	NR	NR	NR	NR
Chavez-MacGregor 2022	14287	NR	NR	NR	NR	NR	NR	NR	NR	NR	NR	NR	NR	NR	NR	NR	NR	NR
Abuhelwa 2022	27760	NR	13495	NR	NR	3340	NR	NR	NR	NR	NR	1675	NR	NR	4400	NR	4080	NR
Serraino 2021	466	NR	NR	NR	NR	NR	NR	NR	NR	NR	NR	NR	NR	NR	NR	NR	NR	NR
Kim 2022	10426	2224	8952	1580	NR	2143	NR	291	476	207	474	794	NR	409	887	NR	1781	NR
Zhou 2023	142	NR	NR	NR	NR	27	NR	NR	NR	NR	NR	11	NR	NR	9	14	8	85
Raad 2023	1115	NR	NR	NR	NR	NR	NR	NR	NR	NR	NR	NR	NR	NR	NR	NR	NR	NR
Rugge 2022	324	41	NR	NR	NR	34	NR	NR	NR	NR	NR	34	NR	NR	23	NR	63	129
Anantharaman 2021	33	NR	NR	NR	NR	NR	NR	NR	NR	NR	NR	NR	NR	NR	NR	NR	NR	NR
Johannesen 2021	53	NR	NR	NR	NR	NR	NR	NR	NR	NR	NR	NR	NR	NR	NR	NR	NR	NR

Sng 2020	94	NR	NR	19	58	8	NR	9	NR	NR	NR	NR	NR	NR	15	NR	24	NR
Alpert 2021	421	NR	NR	NR	NR	NR	NR	NR	NR	NR	NR	NR	NR	NR	NR	NR	NR	NR
Bertuzzi 2021	46	NR	NR	NR	NR	NR	NR	NR	NR	NR	NR	NR	NR	NR	NR	NR	NR	NR
Brar 2020	117	NR	NR	NR	NR	NR	NR	NR	NR	NR	NR	NR	NR	NR	NR	NR	NR	NR
Klein 2021	77	NR	NR	NR	NR	NR	NR	NR	NR	NR	NR	NR	NR	NR	NR	NR	NR	NR
Dai 2020	31	NR	NR	12	15	NR	NR	NR	NR	NR	NR	NR	NR	NR	31	NR	NR	NR
**Total**	281270	8174	61324	1611	73	9635	47	2097	1174	278	745	20143	0	534	7146	146	11144	653
**Percentage**		2.9	21.8	0.6	0	3.4	0	0.7	0.4	0.1	0.3	7.2	0	0.2	2.5	0.1	4	0.2

NR, Not reported.

Table S11: Breakdown of cancer treatments across the 30 included studies

Reference	Total patients with SARS-CoV-2 and cancer (n)	Patients on recent cancer treatments (n)					
		Chemotherapy	Hormonal Therapy	Immunotherapy	Radiotherapy	Surgery	Targeted Therapy
Alsakarneh 2024	15416	4958	1011	10	1418	NR	1837
Li 2024	77	43	NR	NR	NR	NR	NR
Starkey 2023	127322	NR	NR	NR	NR	NR	NR
Konermann 2023	1513	NR	NR	NR	NR	NR	NR
Park 2024	40334	NR	NR	NR	NR	NR	NR
Turtle 2023	5116	NR	NR	NR	NR	NR	NR
Salvatore 2023	6143	1705	NR	NR	NR	NR	NR
Leuva 2022	10355	NR	NR	NR	NR	NR	NR
Hosseini-Moghaddam 2023	8378	NR	NR	NR	NR	NR	NR
Sullivan 2023	2200	491	1347	109	189	181	NR
Nolan 2023	7141	NR	NR	NR	NR	NR	NR
Kodde 2023	1625	NR	NR	NR	NR	NR	NR
Udovica 2022	89	NR	NR	NR	NR	NR	NR
Bazgir 2022	64	27	NR	NR	NR	NR	NR
Plais 2022	105	NR	NR	NR	NR	NR	NR
Chavez-MacGregor 2022	14287	NR	NR	NR	NR	NR	NR
Abuhelwa 2022	27760	NR	NR	NR	NR	NR	NR
Serraino 2021	466	NR	NR	NR	NR	NR	NR
Kim 2022	10426	NR	NR	NR	NR	NR	NR
Zhou 2023	142	NR	NR	NR	NR	NR	NR
Raad 2023	1115	NR	NR	NR	NR	NR	NR
Rugge 2022	324	NR	NR	NR	NR	NR	NR
Anantharaman 2021	33	NR	NR	NR	NR	NR	NR
Johannesen 2021	53	NR	NR	NR	NR	NR	NR
Sng 2020	94	15	8	4	5	3	2
Alpert 2021	421	NR	NR	NR	NR	NR	NR
Bertuzzi 2021	46	NR	NR	NR	NR	NR	NR
Brar 2020	117	NR	NR	NR	NR	NR	NR
Klein 2021	77	NR	NR	NR	NR	NR	NR
Dai 2020	31	NR	NR	NR	NR	NR	NR
Total	281270	7239	2366	123	1612	184	1839

NR, Not reported.

Supplementary Figure 1 - Geographical distribution of cancer patients across 30 studies

Choropleth depicting geographical distribution of patients with cancer. The darker a country, the more patients are from the country. Brazil, Spain, Lebanon, Singapore, Australia, and Japan and represented by NR as Raad 2023 does not report recruitment data from each participating study centre.

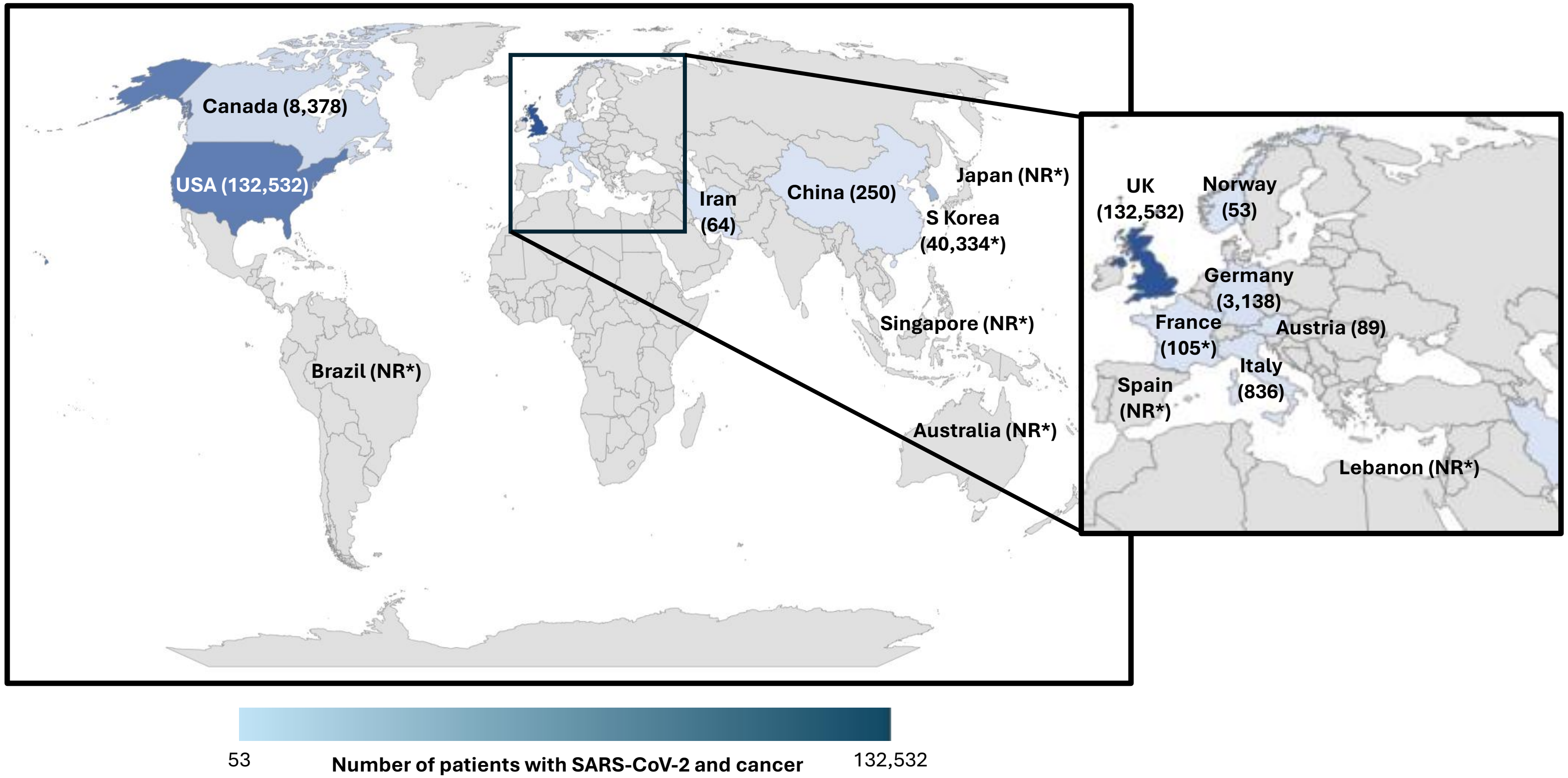

NR, Not reported; *, It was not possible to include patient numbers from Raad et al. 2023 as it was a multinational study that did not stratify cancer patients by country of origin.

Supplementary Figure 2 - Geographical distribution of the 30 included studies

Choropleth depicting geographical distribution of included studies. The darker a country, the more studies included the country.

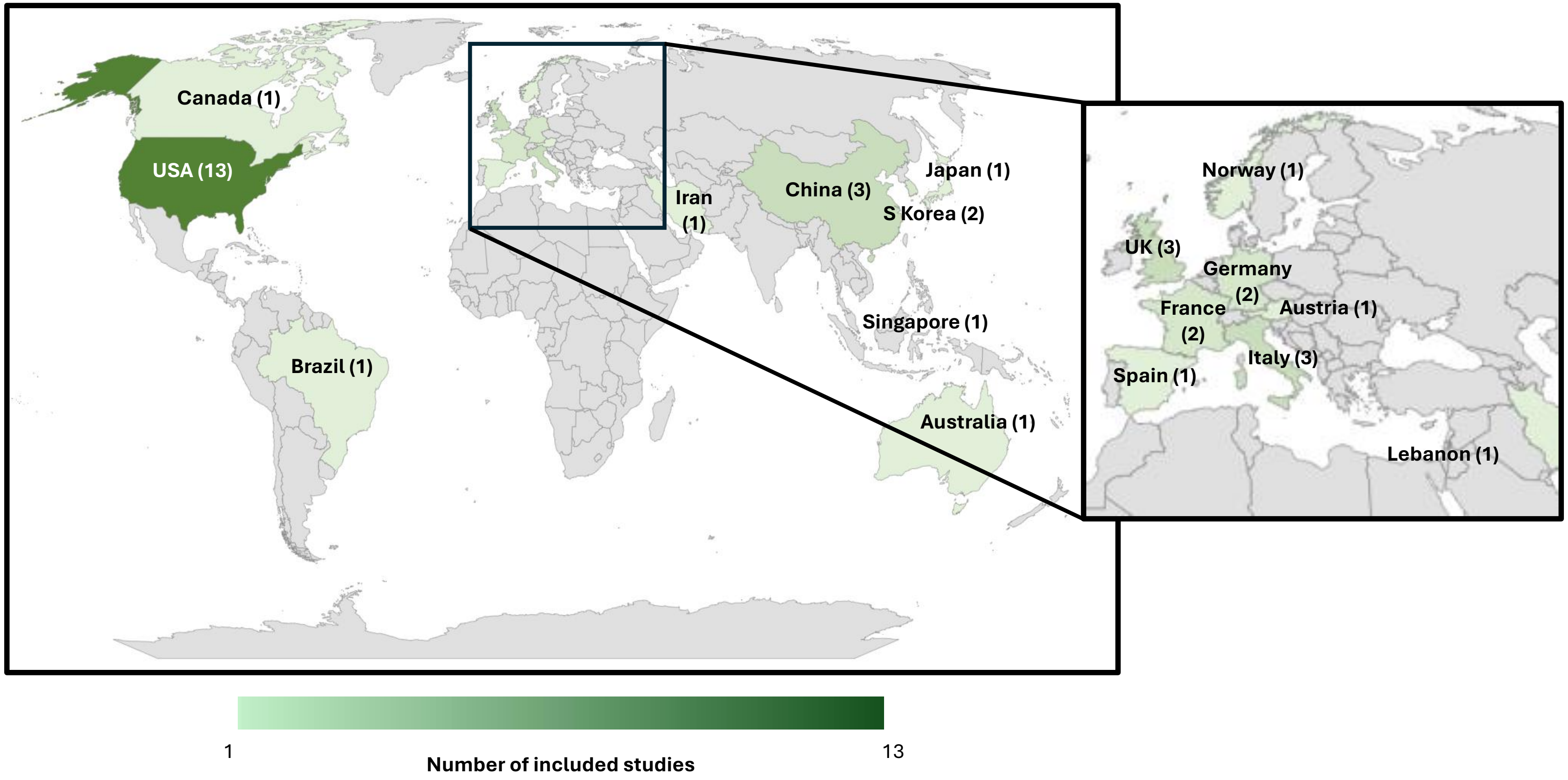

Supplementary Figure 3 - Heatmap showing percentage of comorbidities in included studies' cancer and control cohorts

The darker the study shading, the more prevalent the comorbidity. Gray tiles represent the comorbidity in the patient group is not reported, or only reported in the total cancer cohort, rather than the active cancer cohort of interest. CVD, cardiovascular disease; CKD, chronic kidney disease.

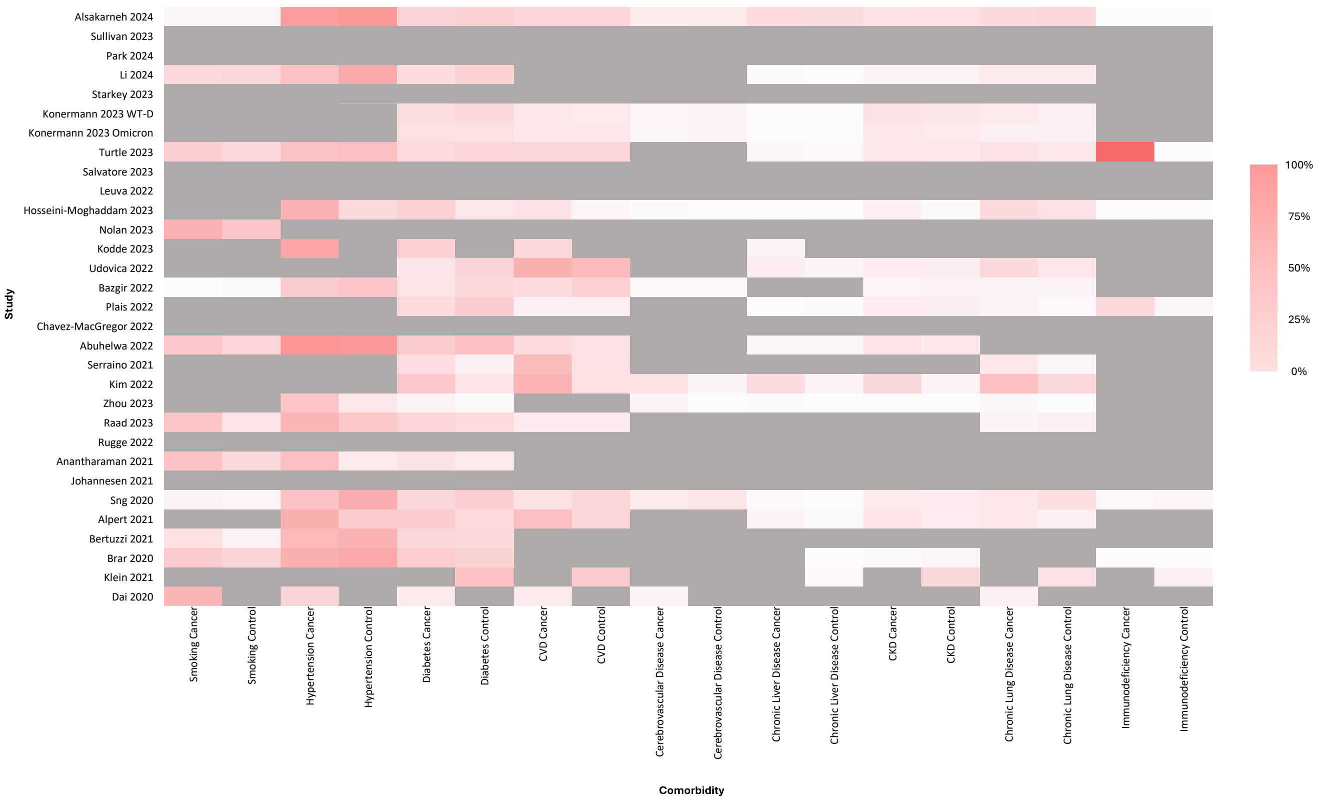

Supplementary Figure 4 - Number of patients and number of studies by cancer types for studies which included a breakdown

The height of the bar graphs represent the total number of patients with cancer with that particular cancer type, whilst the label indicates the number of studies that reported numbers of patients with cancer for that respective cancer type.

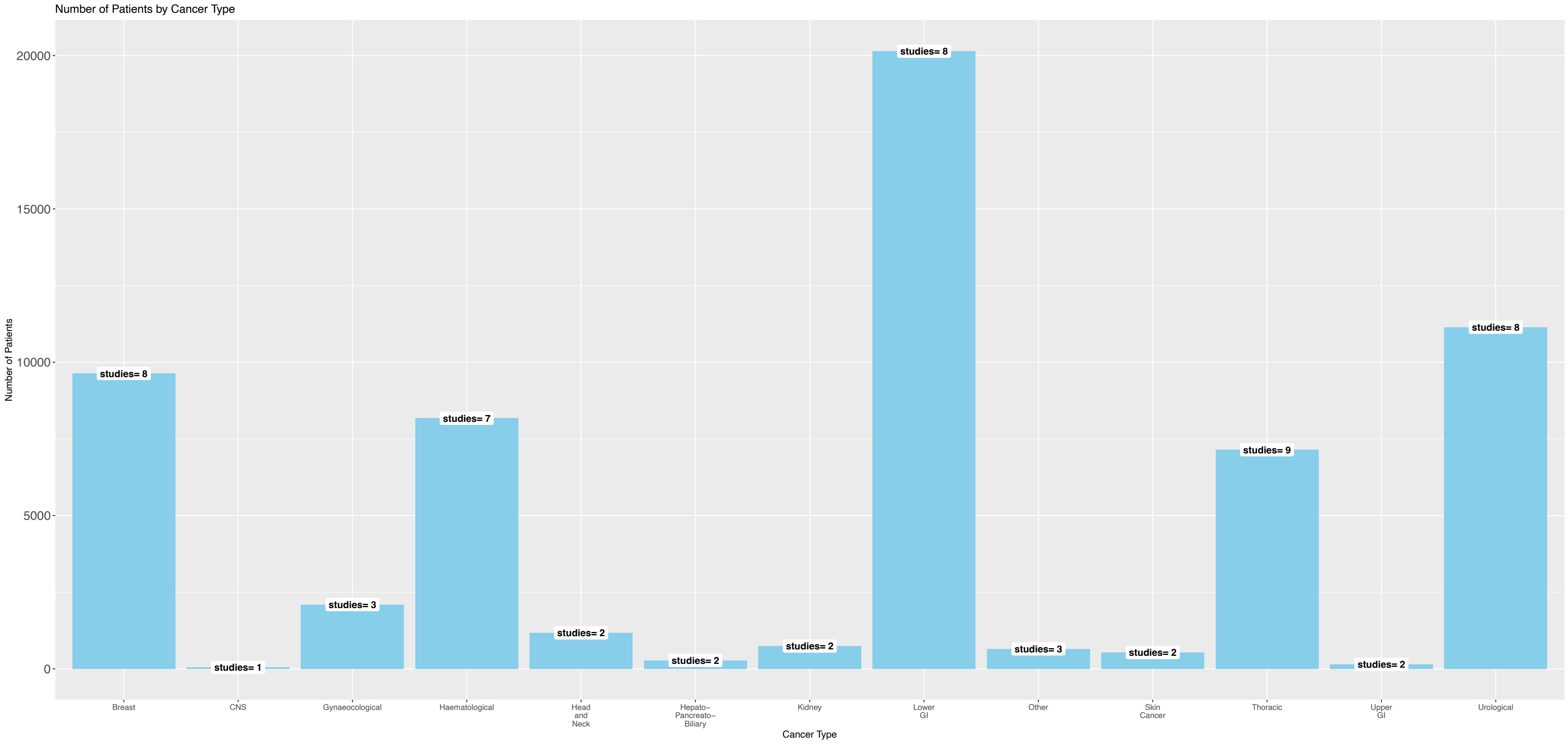

Supplementary Figure 5 - Number of patients and studies which describe different forms of anti-cancer therapy for studies which provided a breakdown

The height of the bar graphs represent the total number of patients with cancer with that particular cancer type, whilst the label indicates the number of studies that reported numbers of patients with cancer for that respective cancer type.

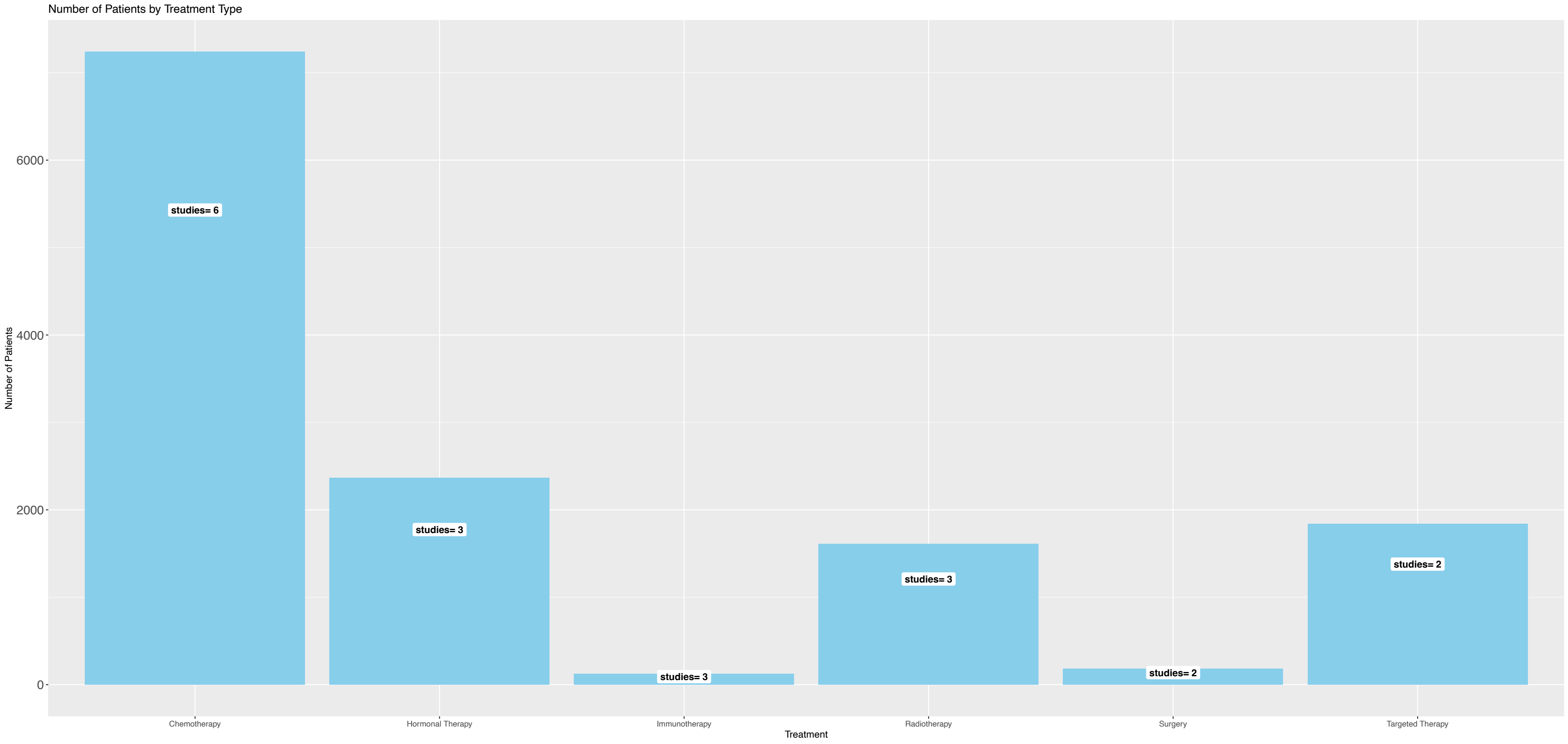

Supplementary Figure 6 - Total number of sequences queried via LAPIS and Outbreak.info stratified by study
Total number of sequences queried per study (logarithmic scale) on NCBI Genbank via LAPIS API (red) and GISAID via Outbreak info API (blue).

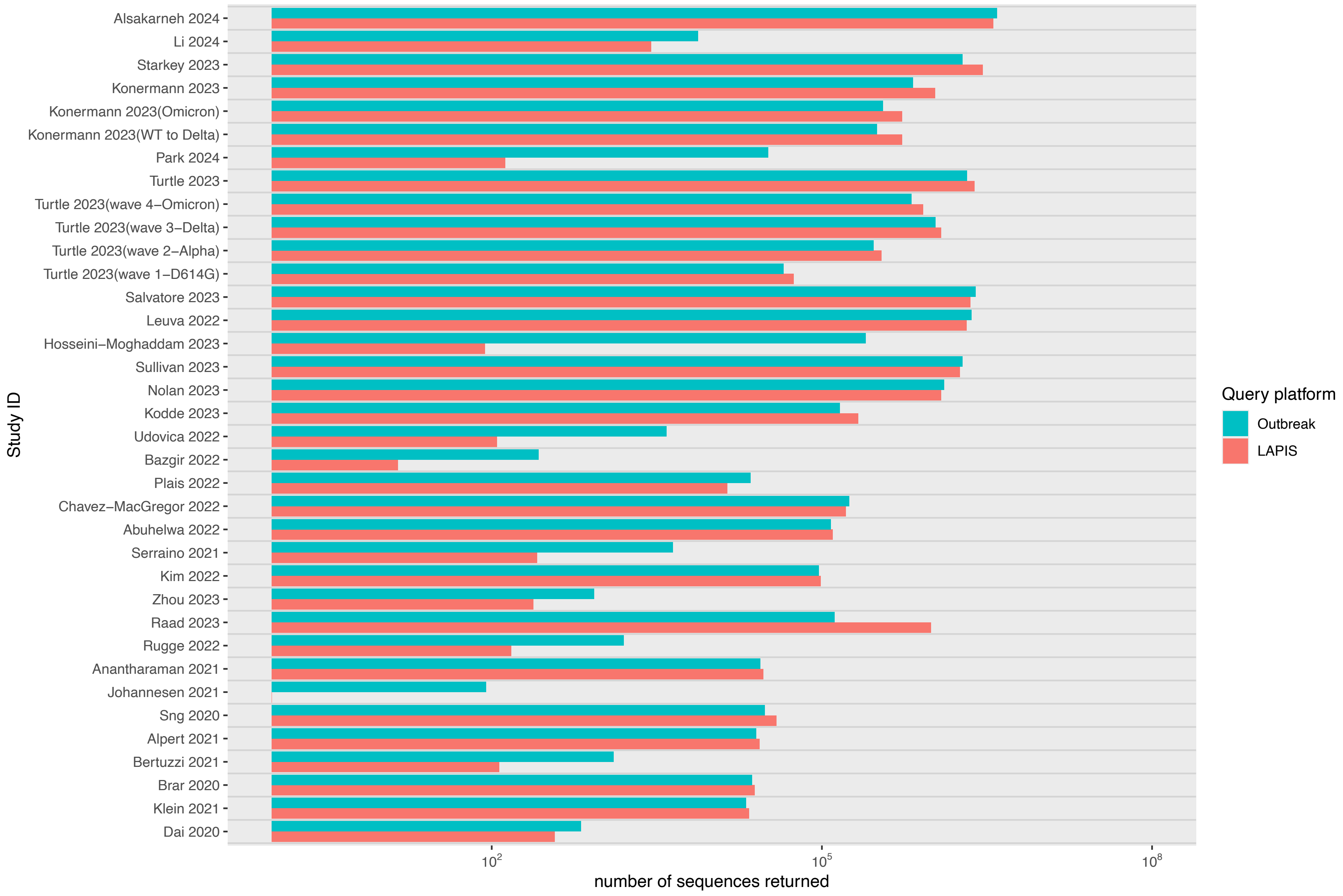

Supplementary Figure 7 - Total number of sequences queried via LAPIS and Outbreak.info stratified by country

Total number of sequences queried per country (logarithmic scale) on NCBI Genbank via LAPIS API (red) and GISAID via Outbreak info API (blue).

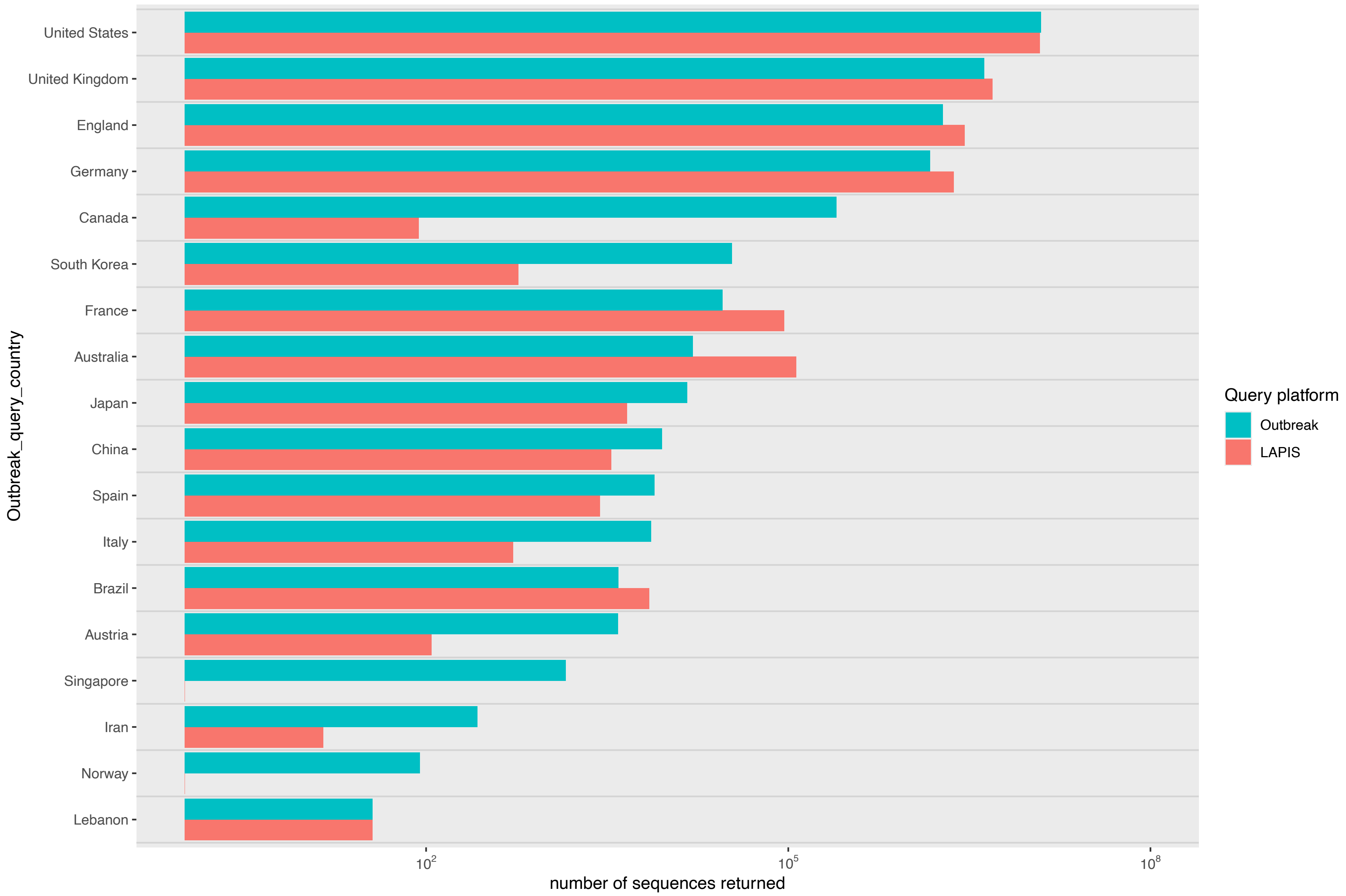

Supplementary Figure 8 - Relationship between COVID-19 case incidence, death incidence, mortality rate, and sequencing performed for each study
Total number of WHO reported new SARS-CoV-2 cases (black), new SARS-CoV-2 deaths (red) and deaths per 1000 cases (dark red), NCBI Genbank (via LAPIS) queried sequences (green) and queried sequences per 1000 cases (dark green), and GISAID (via Outbreak.info) sequences (blue) and sequences per 1000 cases (dark blue), stratified by study period and country

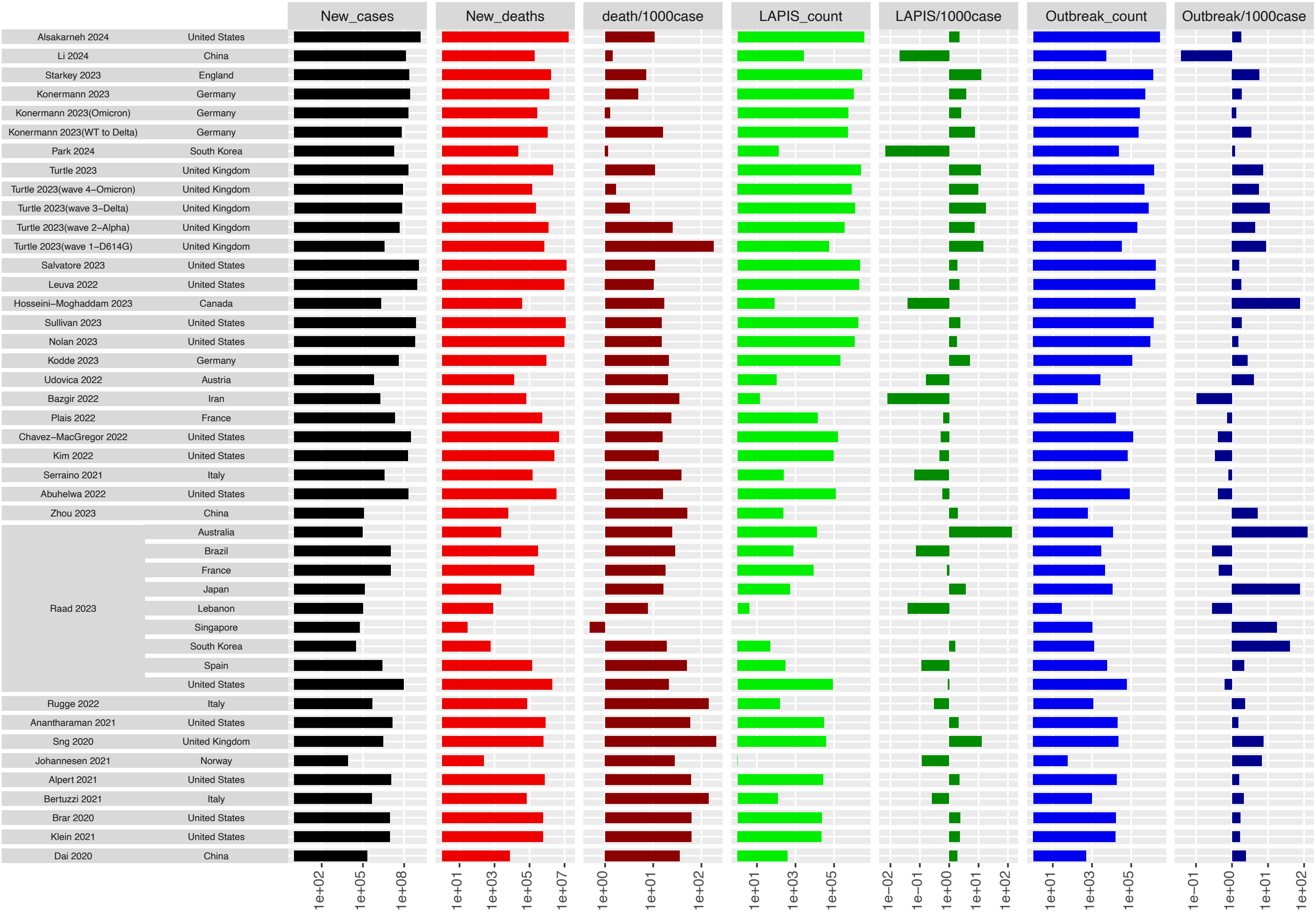

Supplementary Figure 9 - Genomic imputation for the multinational study Raad 2023

The weekly evolution of major variants reported from LAPIS (top) and Outbreak.info (middle), and log transformed 4-week simple moving average of WHO new cases (bottom, black) and new deaths (bottom, red) in the Multinational study Raad 2023.

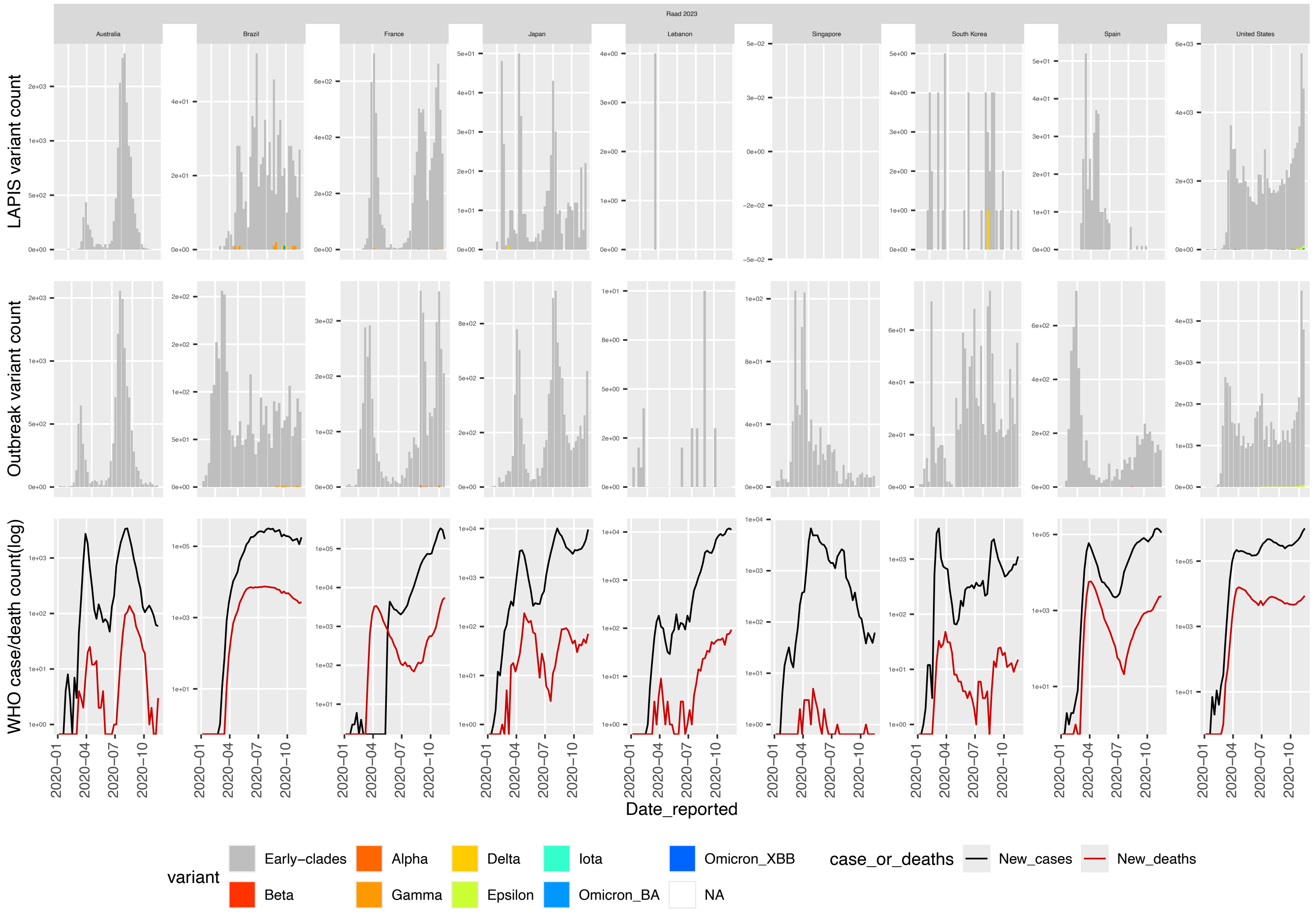

Supplementary Figure 10 - Genomic imputation for studies with a VOC majority

The weekly evolution of major variants reported from LAPIS (top) and Outbreak.info (middle), and log transformed 4-week moving average of WHO new cases (bottom, black) and new deaths (bottom, red) in studies which reported dominance of a VOC.

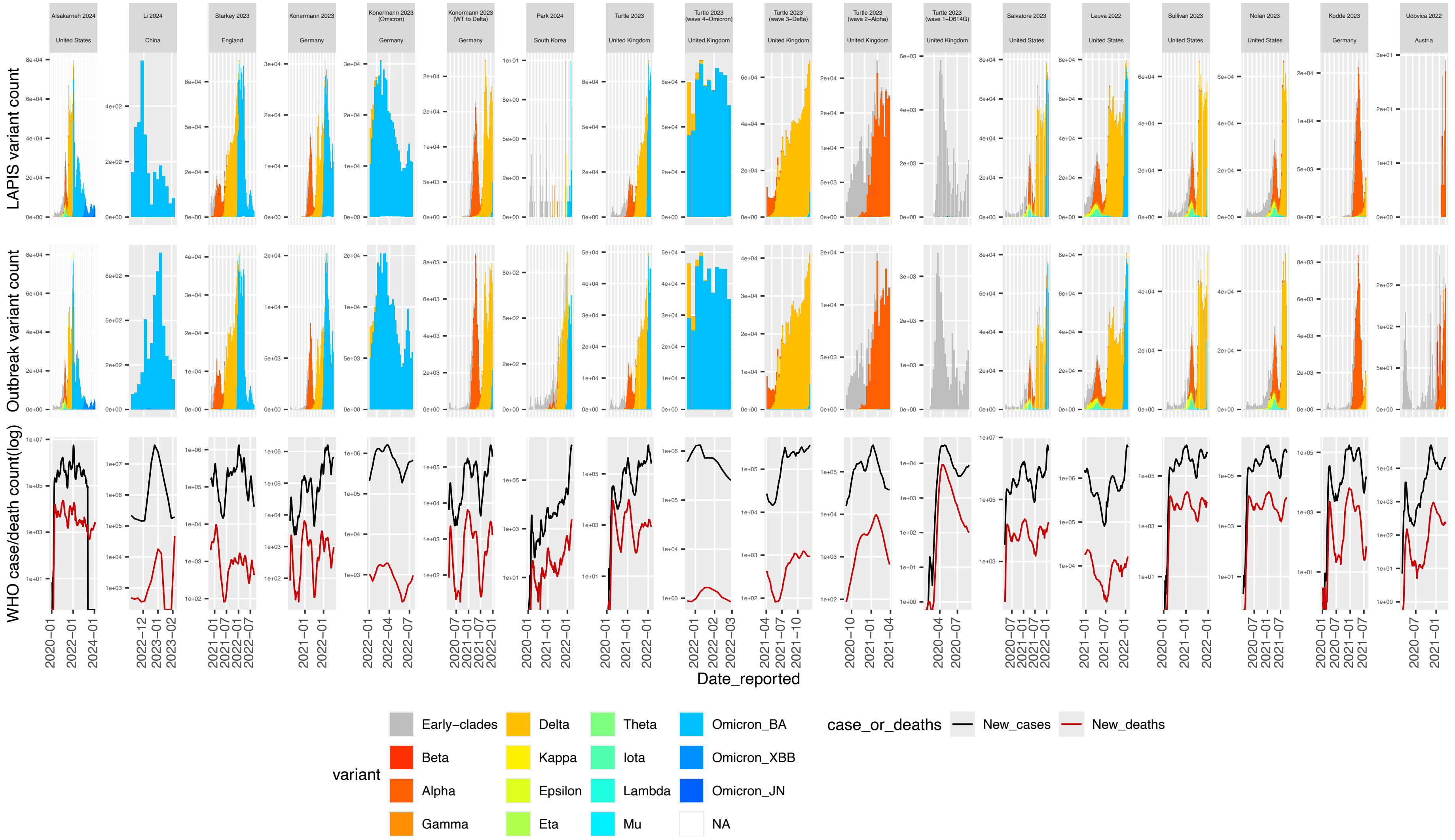

Supplementary Figure 11a - Pooled odds ratios of hospitalisation, severe disease, ICU admission, and mortality in COVID-19 patients with any type of cancers compared with those without cancer

Multivariate hospitalisation, severe disease, ICU admission, and mortality odds ratio of all cancers compared to non-cancer controls.

Pooled Odds Ratios of Hospitalisation, ICU Admission, Mortality and Severe Disease Amongst the Total Cancer Cohort versus Controls

<-- Greater Odds In Control Patients | Greater Odds In Cancer Patients -->

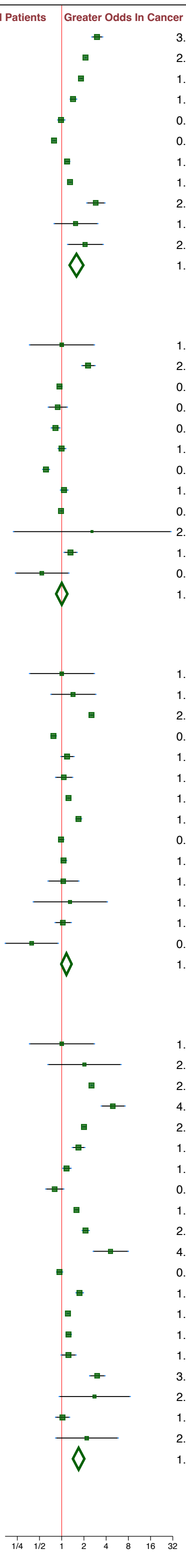

Study	OR with 95% CI	Weight	Cancer (n)	Non-Cancer (n)
**Hospitalisation**				
Alsakarneh 2024 (Total Cancer Cohort)	3.01 [2.55, 3.56]	9.50%	15,416	15,416
Starkey 2023 (Total Cancer Cohort)	2.10 [2.07, 2.14]	9.84%	127,322	15,801,004
Park 2024 (Total Cancer Cohort)	1.82 [1.73, 1.91]	9.81%	397,050	397,050
Salvatore 2023 (Total Cancer Cohort)	1.43 [1.29, 1.58]	9.72%	6,143	38,267
Sullivan 2023 (Total Cancer Cohort)	0.98 [0.88, 1.09]	9.69%	2,200	22,000
Chavez-MacGregor 2022 (Total Cancer Cohort) *	0.79 [0.75, 0.83]	9.82%	9,991	493,020
Chavez-MacGregor 2022 (Total Cancer Cohort) *	1.19 [1.11, 1.27]	9.79%	4,296	493,020
Kim 2022 (Total Cancer Cohort)	1.30 [1.24, 1.36]	9.82%	10,426	253,179
Rugge 2022 (Total Cancer Cohort)	2.88 [2.17, 3.82]	8.91%	263	20,047
Anantharaman 2021 (Total Cancer Cohort)	1.55 [0.78, 3.06]	6.10%	33	4,380
Johannesen 2021 (Total Cancer Cohort)	2.08 [1.19, 3.62]	7.00%	53	7,841
Heterogeneity: $\chi^2(10) = 2071.1$, $p < 0.0001$, I^2=99.5%	1.58 [1.22, 2.06]			
H_0: OR = 1, z = 3.46, $p = 0.0005$				
**Severe Disease**				
Alsakarneh 2024 (Total Cancer Cohort)	1.00 [0.36, 2.75]	4.35%	15,416	15,416
Park 2024 (Total Cancer Cohort)	2.29 [1.87, 2.81]	9.70%	397,050	397,050
Turtle 2023 (Total Cancer Cohort)	0.93 [0.86, 1.00]	10.16%	5,116	134,598
Sullivan 2023 (Total Cancer Cohort)	0.88 [0.66, 1.18]	9.18%	2,200	22,000
Kodde 2023 (Total Cancer Cohort)	0.82 [0.72, 0.94]	10.00%	1,625	27,659
Chavez-MacGregor 2022 (Total Cancer Cohort) *	1.00 [0.89, 1.12]	10.05%	4,296	493,020
Chavez-MacGregor 2022 (Total Cancer Cohort) *	0.61 [0.55, 0.68]	10.09%	9,991	493,020
Kim 2022 (Total Cancer Cohort)	1.07 [0.96, 1.20]	10.06%	10,426	253,179
Abuhelwa 2022 (Total Cancer Cohort)	0.98 [0.94, 1.02]	10.21%	27,760	1,022,285
Anantharaman 2021 (Total Cancer Cohort)	2.57 [0.22, 29.97]	1.14%	33	4,380
Alpert 2021 (Total Cancer Cohort)	1.31 [1.07, 1.60]	9.71%	421	5,135
Klein 2021 (Total Cancer Cohort)	0.54 [0.24, 1.24]	5.35%	77	324
Heterogeneity: $\chi^2(11) = 164.7$, $p < 0.0001$, I^2=97.0%	1.00 [0.80, 1.25]			
H_0: OR = 1, z = 0.02, $p = 0.98$				
**ICU Admission**				
Alsakarneh 2024 (Total Cancer Cohort)	1.00 [0.36, 2.75]	3.72%	15,416	15,416
Li 2024 (Total Cancer Cohort)	1.43 [0.71, 2.88]	5.31%	77	1,788
Starkey 2023 (Total Cancer Cohort)	2.53 [2.38, 2.68]	8.71%	127,322	15,801,004
Turtle 2023 (Total Cancer Cohort)	0.77 [0.71, 0.84]	8.67%	5,116	134,598
Salvatore 2023 (Total Cancer Cohort)	1.19 [0.97, 1.46]	8.29%	6,143	38,267
Sullivan 2023 (Total Cancer Cohort)	1.07 [0.82, 1.39]	8.01%	2,200	22,000
Nolan 2023 (Total Cancer Cohort)	1.24 [1.17, 1.31]	8.71%	7,141	90,700
Chavez-MacGregor 2022 (Total Cancer Cohort) *	1.69 [1.55, 1.85]	8.66%	4,296	493,020
Chavez-MacGregor 2022 (Total Cancer Cohort) *	0.98 [0.91, 1.06]	8.68%	9,991	493,020
Kim 2022 (Total Cancer Cohort)	1.06 [0.98, 1.15]	8.67%	10,426	253,179
Rugge 2022 (Total Cancer Cohort)	1.05 [0.65, 1.70]	6.70%	1,787	20,777
Anantharaman 2021 (Total Cancer Cohort)	1.30 [0.41, 4.12]	3.17%	33	4,380
Alpert 2021 (Total Cancer Cohort)	1.04 [0.81, 1.34]	8.08%	421	5,135
Klein 2021 (Total Cancer Cohort)	0.39 [0.17, 0.89]	4.61%	77	324
Heterogeneity: $\chi^2(13) = 763.4$, $p < 0.0001$, I^2=97.3%	1.16 [0.94, 1.42]			
H_0: OR = 1, z = 1.41, $p = 0.16$				
**Mortality**				
Alsakarneh 2024 (Total Cancer Cohort)	1.00 [0.36, 2.75]	2.53%	15,416	15,416
Li 2024 (Total Cancer Cohort)	2.02 [0.65, 6.32]	2.19%	77	1,788
Starkey 2023 (Total Cancer Cohort)	2.54 [2.49, 2.60]	5.96%	127,322	15,801,004
Park 2024 (Total Cancer Cohort)	4.95 [3.42, 7.17]	5.05%	397,050	397,050
Turtle 2023 (Total Cancer Cohort)	2.00 [1.89, 2.12]	5.94%	5,116	134,598
Salvatore 2023 (Total Cancer Cohort)	1.68 [1.38, 2.04]	5.68%	6,143	38,267
Leuva 2022 (Total Cancer Cohort)	1.17 [1.03, 1.33]	5.84%	10,355	10,696
Sullivan 2023 (Total Cancer Cohort)	0.80 [0.60, 1.06]	5.41%	2,200	22,000
Nolan 2023 (Total Cancer Cohort)	1.58 [1.47, 1.69]	5.93%	7,141	90,700
Kodde 2023 (Total Cancer Cohort)	2.10 [1.88, 2.34]	5.87%	1,625	27,659
Udovica 2022 (Total Cancer Cohort)	4.59 [2.66, 7.92]	4.28%	89	156
Chavez-MacGregor 2022 (Total Cancer Cohort) *	0.93 [0.85, 1.02]	5.90%	9,991	493,020
Chavez-MacGregor 2022 (Total Cancer Cohort) *	1.74 [1.55, 1.95]	5.87%	4,296	493,020
Abuhelwa 2022 (Total Cancer Cohort)	1.21 [1.17, 1.25]	5.96%	27,760	1,022,285
Kim 2022 (Total Cancer Cohort)	1.24 [1.15, 1.34]	5.92%	10,426	253,179
Raad 2023 (Total Cancer Cohort)	1.23 [0.98, 1.55]	5.57%	1,115	2,851
Rugge 2022 (Total Cancer Cohort)	3.01 [2.38, 3.82]	5.55%	324	20,777
Anantharaman 2021 (Total Cancer Cohort)	2.78 [0.92, 8.40]	2.28%	33	4,380
Alpert 2021 (Total Cancer Cohort)	1.02 [0.82, 1.27]	5.62%	421	5,135
Klein 2021 (Total Cancer Cohort)	2.18 [0.82, 5.77]	2.65%	77	324
Heterogeneity: $\chi^2(19) = 2048.6$, $p < 0.0001$, I^2=99.2%	1.70 [1.36, 2.12]			
H_0: OR = 1, z = 4.63, $p < 0.0001$				

Restricted maximum-likelihood estimates for the pooled OR, weighted by the inverse variance of reported multivariate-adjusted ORs.
H_0: OR = 1 refers to the null hypothesis that the pooled OR for a given outcome is equal to 1.
Heterogeneity is reported both as the Cochran's Q statistic, χ^2, and the I^2 statistic.
The p value in the heterogeneity is derived from the Cochran's Q statistic, i.e., the likelihood that the individual studies in each sub-group report multivariate-adjusted ORs sampled from one homogeneous population.
* The cancer cohort was split into recent treatment (n=4,296) and no recent treatment (n=9,991)

Supplementary Figure 11b - Pooled hazard ratios of ICU admission and mortality in COVID-19 patients with any type of cancers compared with those without cancer

Multivariate ICU admission, and mortality hazard ratio of all cancers compared to non-cancer controls. There were insufficient studies for hospitalisation and severe disease for pooled hazard ratio analysis.

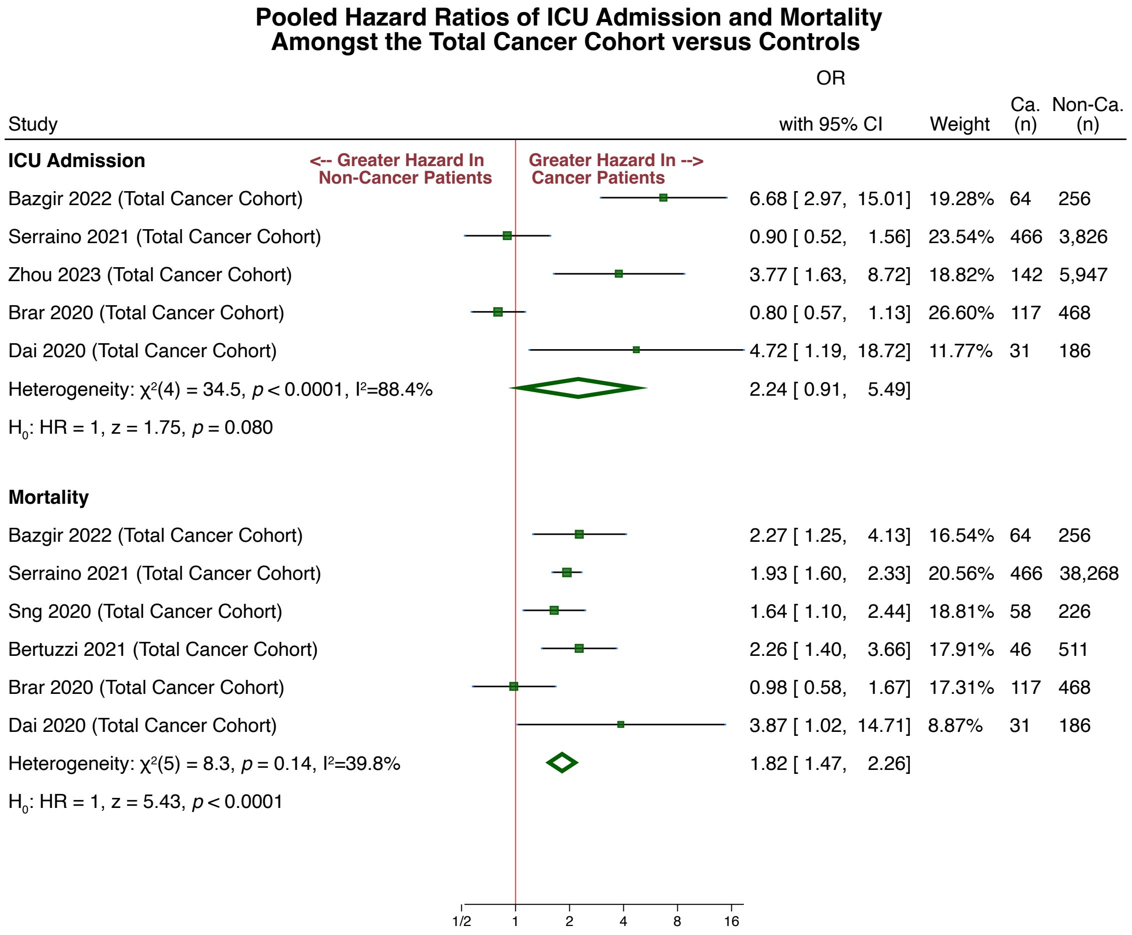

Restricted maximum-likelihood estimates for the pooled HR, weighted by the inverse variance of reported multivariate-adjusted HRs.
H_0: HR = 1 refers to the null hypothesis that the pooled HR for a given outcome is equal to 1.
Heterogeneity is reported both as the Cochran's Q statistic, χ^2, and the I^2 statistic.
The p value in the heterogeneity is derived from the Cochran's Q statistic, i.e., the likelihood that the individual studies in each sub-group report multivariate-adjusted HRs sampled from one homogeneous population.

Supplementary Figure 12 - Pooled odds ratio of mortality in patients with cancer compared to patients without cancer of different vaccination status. Multivariate mortality odds ratio stratified by overall vaccination status of the study population. Studies are split by if less than 50% of patients have received a full primary course of vaccination, more than 50% have received full primary course of vaccination, or if there are no statement on vaccination status

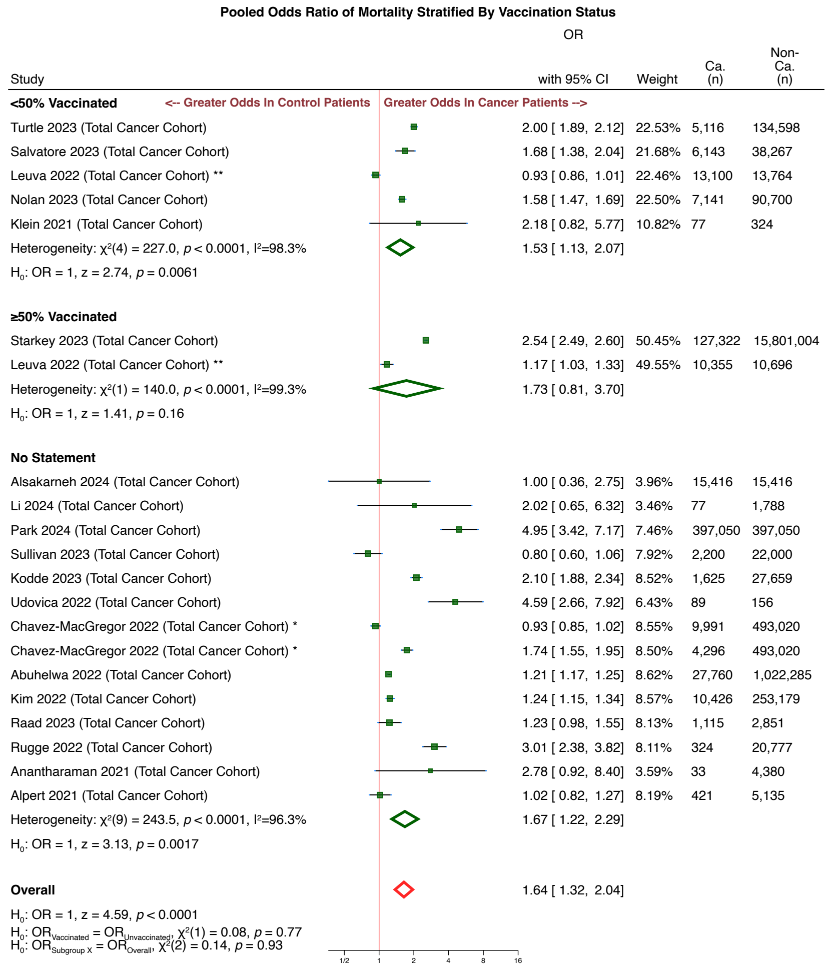

Restricted maximum-likelihood estimates for the pooled OR, weighted by the inverse variance of included studies.
H_0: OR = 1 refers to the null hypothesis that the pooled OR for a given sub-group is equal to 1.
H_0: $OR_{Vaccinated} = OR_{Unvaccinated}$ refers to the null hypothesis that the pooled ORs from the ≥50% and <50% vaccinated cohorts were equal.
H_0: $OR_{Subgroup\ X} = OR_{Overall}$ refers to the null hypothesis that the pooled ORs of each of the three cohorts were equal.
This was assessed using the χ^2 test with N-1 degrees of freedom, where N is the number of sub-groups.
Heterogeneity is reported both as the Cochran's Q statistic, χ^2, and the I^2 statistic.
The p value in the heterogeneity is derived from the Cochran's Q statistic, i.e., the likelihood that the individual studies in each sub-group report multivariate-adjusted ORs sampled from one homogeneous population.
* The cancer cohort of Chavez-MacGregor 2022 was split into recent treatment (n=4,296) and no recent treatment (n=9,991)
** Leuva 2022 provided separate ORs of mortality for the vaccinated and unvaccinated cohorts

Supplementary Figure 13 - AHRQ scores of study quality based on Newcastle-Ottawa Quality Assessment Scale assessment. Proportion of studies with poor (red, n= 8), fair (yellow, n= 1), and good (green, n= 21) quality are plotted as a stacked bar graph.

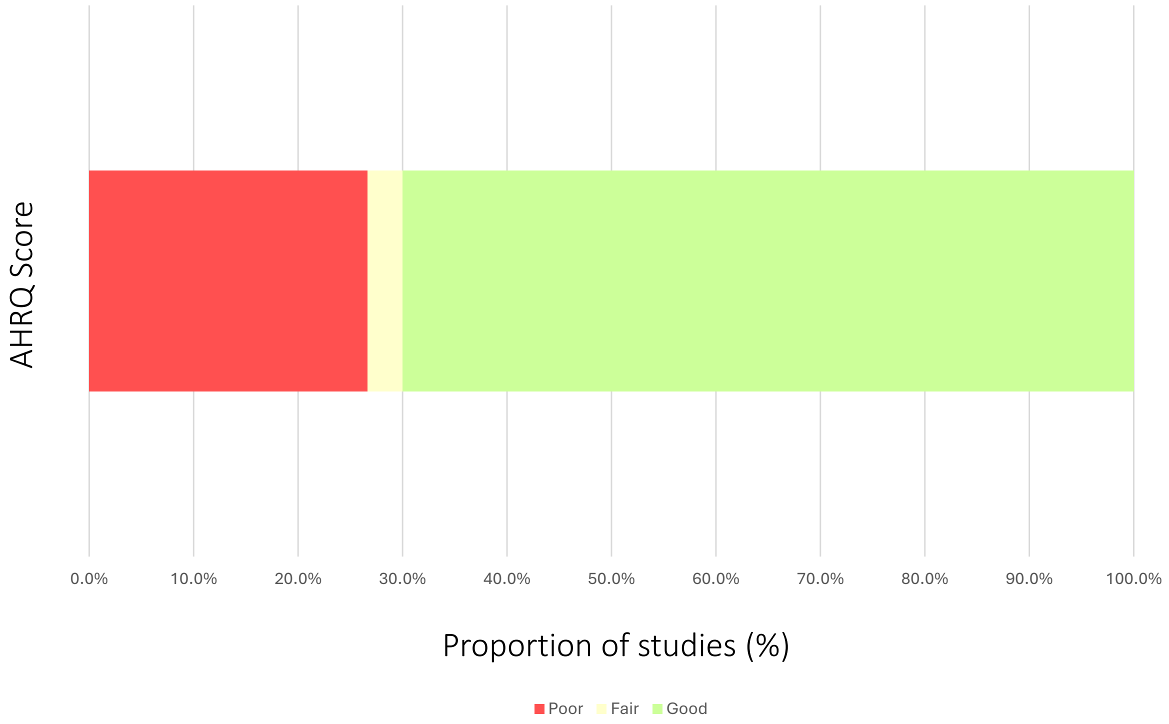

Supplement: Supplementary Figures and Tables [file mmc1.pdf]
